# Comparative whole-genome and proteomics analyses of the next seed bank and the original master seed bank of MucoRice-CTB 51A line, a rice-based oral cholera vaccine

**DOI:** 10.1186/s12864-020-07355-7

**Published:** 2021-01-19

**Authors:** Ai Sasou, Yoshikazu Yuki, Ayaka Honma, Kotomi Sugiura, Koji Kashima, Hiroko Kozuka-Hata, Masanori Nojima, Masaaki Oyama, Shiho Kurokawa, Shinichi Maruyama, Masaharu Kuroda, Shinjiro Tanoue, Narushi Takamatsu, Kohtaro Fujihashi, Eiji Goto, Hiroshi Kiyono

**Affiliations:** 1 Division of Mucosal Immunology, IMSUT Distinguished Professor Unit, The Institute of Medical Science, The University of Tokyo, Tokyo, Japan; 2 Asahi Kogyosha Co., Ltd., Tokyo, Japan; 3 Medical Proteomics Laboratory, The Institute of Medical Science, The University of Tokyo, Tokyo, Japan; 4 Center for Translational Research, IMSUT Hospital, The Institute of Medical Science, The University of Tokyo, Tokyo, Japan; 5 Crop Development Division, NARO Agriculture Research Center, Niigata, Japan; 6 Astellas Pharma Inc., Tokyo, Japan; 7 Research and Development Center for Mucosal Vaccines, The Institute of Medical Science, The University of Tokyo, Tokyo, Japan; 8 Faculty of Horticulture, Graduate School of Horticulture, Chiba University, Chiba, Japan; 9 Department of Immunology, Graduate School of Medicine, Chiba University, Chiba, Japan; 10 Chiba University-University of California San Diego Center for Mucosal Immunology, Allergy, and Vaccine, Division of Gastroenterology, Department of Medicine, University of California, San Diego, California USA

**Keywords:** Plant-made pharmaceuticals, Oral cholera vaccine, Whole-genome re-sequencing, Transgenic rice, Proteomics analysis, Seed bank, MucoRice-CTB, Shot-gun MS/MS

## Abstract

**Background:**

We have previously developed a rice-based oral vaccine against cholera diarrhea, MucoRice-CTB. Using *Agrobacterium*-mediated co-transformation, we produced the selection marker–free MucoRice-CTB line 51A, which has three copies of the cholera toxin B subunit (CTB) gene and two copies of an RNAi cassette inserted into the rice genome. We determined the sequence and location of the transgenes on rice chromosomes 3 and 12. The expression of alpha-amylase/trypsin inhibitor, a major allergen protein in rice, is lower in this line than in wild-type rice. Line 51A was self-pollinated for five generations to fix the transgenes, and the seeds of the sixth generation produced by T5 plants were defined as the master seed bank (MSB). T6 plants were grown from part of the MSB seeds and were self-pollinated to produce T7 seeds (next seed bank; NSB). NSB was examined and its whole genome and proteome were compared with those of MSB.

**Results:**

We re-sequenced the transgenes of NSB and MSB and confirmed the positions of the three CTB genes inserted into chromosomes 3 and 12. The DNA sequences of the transgenes were identical between NSB and MSB. Using whole-genome sequencing, we compared the genome sequences of three NSB with three MSB samples, and evaluated the effects of SNPs and genomic structural variants by clustering. No functionally important mutations (SNPs, translocations, deletions, or inversions of genic regions on chromosomes) between NSB and MSB samples were detected. Analysis of salt-soluble proteins from NSB and MSB samples by shot-gun MS/MS detected no considerable differences in protein abundance. No difference in the expression pattern of storage proteins and CTB in mature seeds of NSB and MSB was detected by immuno-fluorescence microscopy.

**Conclusions:**

All analyses revealed no considerable differences between NSB and MSB samples. Therefore, NSB can be used to replace MSB in the near future.

**Supplementary Information:**

The online version contains supplementary material available at 10.1186/s12864-020-07355-7.

## Background

The production of pharmaceutical proteins in plants has become a promising approach because it offers low-cost production, safety owing to the lack of human or animal pathogens, ease of scaling, and capability to produce complex proteins [1, 2]. Since a functional monoclonal antibody was first expressed in tobacco leaves in 1989 [3], the production of many pharmaceutical proteins for human use has been partially shifted from bacterial and mammalian cell culture to plant-based molecular farming [4].

Proteins can be expressed in plants transiently or stably. In transient expression, modified plant viruses or viral vectors integrated into binary vectors are delivered, for example, via *Agrobacterium* (agroinfiltration). Because integration of the transgene into chromosomes is not needed, protein expression usually peaks in less than 7 days post-infiltration [5]. An example of plant-based pharmaceutical production using transient expression is Zmapp, a cocktail of three monoclonal antibodies (13C6, 2G4, 4G7) against the surface glycoprotein of Ebola virus, in *Nicotiana benthamiana* [6, 7]. The United States Food and Drug Administration (FDA) approved Zmapp as an investigational new drug in 2015, allowing the start of clinical trials in Liberia [4]. In stable expression, which also uses agroinfiltration, T-DNA is integrated into the plant genome. An example of a stably expressed protein is recombinant taliglucerase alfa (ELELYSO, Protalix BioTherapeutics) produced in suspension culture of carrot cells for the treatment of Gaucher’s disease [8–10]. The FDA approved ELELYSO in 2012. Another example is the first plant-based monoclonal antibody against HIV-1 (P2G12) produced in tobacco leaves under good manufacturing practices (GMP) in Europe [11]. The first-in-human, double-blind, placebo-controlled, randomized, dose-escalation phase I safety study of single vaginal administration of P2G12 showed no adverse events related to changes in laboratory results, vital signs, and general physical condition in healthy female subjects [11].

We have previously developed MucoRice-CTB, a rice-based oral vaccine against cholera diarrhea [12]. To establish a MucoRice-CTB line for human use, we have used a two-*Agrobacterium* co-transformation system [13]: one *Agrobacterium* transformant carried a T-DNA binary vector with a selection marker and the other one carried a T-DNA binary vector with cholera toxin B subunit (CTB) over-expression and RNAi cassettes to suppress the expression of the storage proteins glutelin and prolamin in rice seeds, so that the expression and accumulation of CTB was enhanced in the endosperm [14]. Using shot-gun MS/MS, we have shown low expression of several rice allergenic proteins such as alpha-amylase/trypsin inhibitor, suggesting that MucoRice-CTB has potential as a safe oral cholera vaccine for clinical application [15]. Among marker-free co-transformants, we selected line 51A with high CTB expression and advanced it to the T6 generation by self-pollination to obtain a homozygous line [16]. We determined the entire sequences of all the transgene inserts and have found that two copies of the CTB over-expression and RNAi cassettes were inserted in tandem into chromosome 3, and a single truncated copy without half of the RNAi cassette was inserted into chromosome 12 [16].

The seeds of the T6 generation produced by T5 plants were defined as the master seed bank (MSB). Using this line, we have produced MucoRice-CTB in a closed hydroponic system for growing transgenic rice plants under GMP [17] and then, after formulation, we conducted a double-blind, randomized, placebo-controlled, three-cohort, dose-escalation, first-in-human phase I study and confirmed the safety, tolerability, and immunogenicity of MucoRice-CTB in humans in 2016 (manuscript submitted).

Because MSB was preserved for a long time, even though it was stored under cold conditions, its renewal is needed for the development of a sustainable seed bank system. We previously determined the criteria for seed bank renewal, which included appearance, confirmation of CTB, germination rate, concentration of CTB protein, biological activity (GM1 ELISA for CTB), fluctuation in proteins other than CTB, CTB gene by PCR, insertion positions in the rice genome (chromosomes 3 and 12), and insertion sequences (chromosomes 3 and 12) [17]. However, it is necessary to investigate whether seeds produced by self-pollinated plants grown from MSB possess almost the same genetic and proteomic quality as MSB seeds. In this study, T6 plants were grown from part of the MSB seeds in our hydroponic GMP facility and were self-pollinated to produce T7 seeds as the next seed bank (NSB). To demonstrate the genetic stability of NSB, we compared it with the original MSB of MucoRice-CTB line 51A by genomic and proteomic analyses.

## Results

### Yield and CTB quantification

**Fig. 1:**
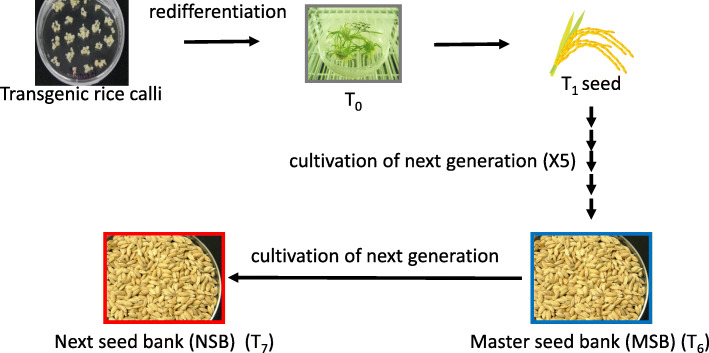
Scheme for generation of MSB and NSB. Rice calli were transformed by *Agrobacterium*. The transgenic line 51A was self-pollinated for five generations to fix the transgene, and the seeds of the sixth generation produced by T5 plants were defined as the master seed bank (MSB). T6 plants were grown from part of the MSB seeds and self-pollinated to produce the next seed bank (NSB; T7 seeds) in a fully closed-type plant production facility built in IMSUT

NSB was produced from MSB in a fully closed-type plant production facility built at The Institute of Medical Science, The University of Tokyo (IMSUT) (Fig. 1). NSB yield was 411.8 g/m^2^, and that of MSB was 387.7 g/m^2^. Average CTB content (μg/mg seed weight) was 6.45 ± 0.89 in MSB and 5.83 ± 0.58 in NSB, with no significant difference (*P* = 0.60). These results suggest that the yield and CTB amounts were very similar between NSB and MSB.

### Confirmation of transgene sequences on chromosomes 3 and 12

**Fig. 2:**
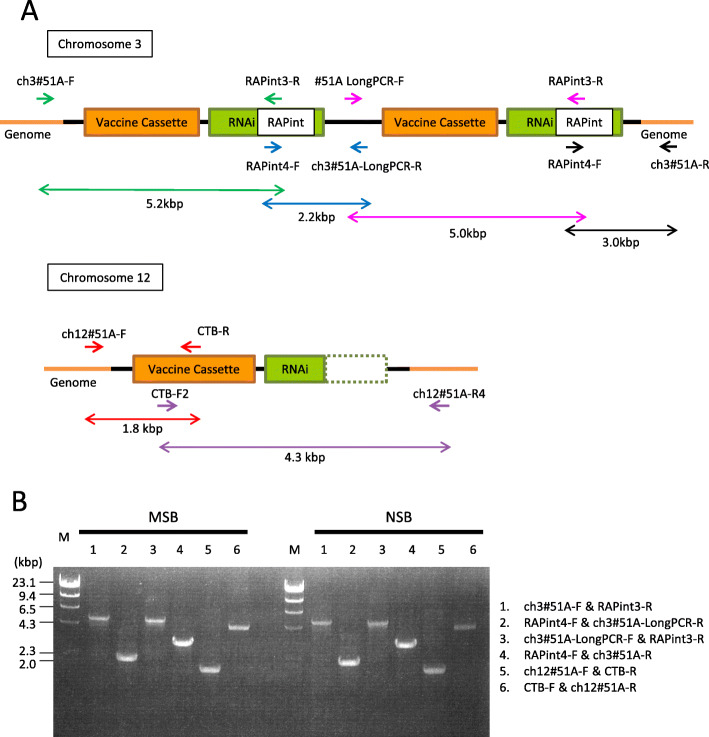
PCR fragments used for re-sequencing of transgenes on chromosomes 3 and 12. **a** Structure of the transgenes, positions of primers. **b** Agarose electrophoresis of the PCR fragments shown in (**a**). All fragments were of the expected size; they were cloned and sequenced

We detected a couple of point mutations in the transgene in NSB in comparison with MSB. To exclude the possibility of PCR errors, we designed PCR primers to amplify a shorter fragment than those we used previously (Additional file 1: Table S1), and also changed TaKaRa LA Taq PCR enzyme to KOD FX. The transgenes were amplified to produce 4 fragments from chromosome 3 and 2 fragments from chromosome 12 (Fig. 2). In comparison with the previous MSB sequence data, sequencing of the PCR fragments identified a single base deletion in a fragment derived from chromosome 3 and five C-to-T substitutions in fragments derived from chromosome 12 (Additional file 2: Figure S1). We repeated this analysis for three seeds picked randomly from each of NSB and MSB. The revised transgene DNA sequences completely matched transgene sequences from all six seeds (Additional file 2: Figure S1). The finding confirms the genetical stability of NSB in comparison with MSB.

### Whole-genome sequencing, clustering analysis, and mRNA analysis

We sequenced the genomic DNA isolated from seedlings grown from three NSB seeds and three MSB seeds. We used different next-generation sequencers: MSB_1 and NSB_1 was sequenced on a HiSeq2000 or HiSeq2500, and NSB_2, NSB_3, MSB_2, and MSB_3 were sequenced on a NovaSeq6000. After filtering to exclude reads with low sequence-quality scores, about 400 million paired-end reads were obtained for MSB_1 and NSB_1 each, and 170 to 210 million pair-end reads each were obtained for NSB_2, NSB_3, MSB_2, and MSB_3. The reads from each sample were aligned separately to the rice reference genome. The mapping rates ranged from 96.72 to 99.24% (Table 1). The coverage rate ranged from 86.2 to 97.3%, whereas the depth (the average number of reads covering a genome) ranged from 56.35 to 118.72.

**Table 1:** Summary of sequence reads for MSB and NSB samples

		Total reads	Mapped reads	Mapping rate (%)	Coverage rate (%)	Depth average	DDBJ experiment accession No.^a^
PG0217_02_a	MSB_1	389,649,421	382,752,568	98.23	94.6	97.54	DRX246636
PG2764_02_a	MSB_2	181,363,917	177,123,344	97.66	93.1	60.12	DRX246637
PG2764_07_b	MSB_3	210,105,305	206,082,524	98.09	97.3	68.83	DRX246638
PG2302_01_a	NSB_1	471,799,917	468,225,863	99.24	95.3	118.72	DRX246639
PG2764_03_a	NSB_2	170,255,253	164,675,845	96.72	86.2	56.35	DRX246640
PG2764_08_b	NSB_3	176,372,027	173,912,788	98.61	91.4	59.29	DRX246641

Mapping rate is the ratio of the number of mapped reads to that of total reads. Covered length is the number of genome bases covered with at least one read. Coverage rate is the ratio of covered length to the total length of the rice reference genome (373,245,519 bps, IRGSP-1.0, build 5 in RAP-DB (https://rapdb.dna.affrc.go.jp/download/irgsp1.html)). Depth (the average number of reads covering a genome) was calculated by dividing the total length of all mapped reads (100 bps per read) by covered length. ^a^ Data set of each experiment accession number is listed in https://www.ncbi.nlm.nih.gov/sra/?term=DRA011151

**Fig. 3:**
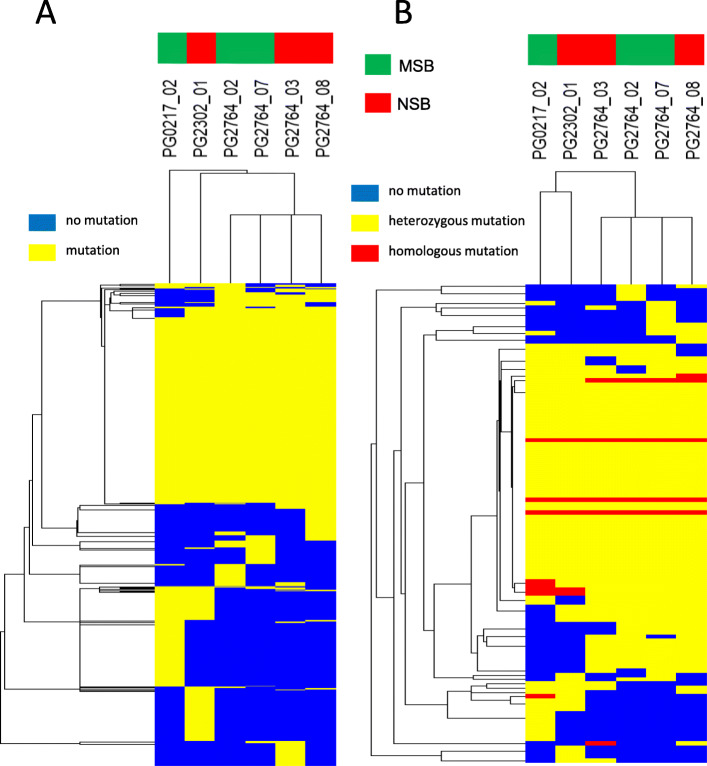
Clustering of mutations in whole-genome sequence analysis. **a** Clustering of SNPs of the genic region on chromosomes. The number of clustered genes was 783. **b** Clustering related to structural changes (translocations, deletions, and inversions) of the genic regions on chromosomes. The number of clustered genes was 112. In both panels, the presence of mutations is indicated relative to the WT reference genome

Next, we carried out cluster analysis of SNPs and structural variants in the genic regions of chromosomes using the gene datasets obtained, because these changes may affect the phenotype. SNPs were detected using mapping results. Structural variants (deletions, inversions, duplications, or translocations) were detected using paired reads showing a different mapping position or different length from the reference DNA sequence. The effect of each detected mutation was evaluated according to the criteria listed in Additional file 1: Table S2, and the genes with a mutation evaluated as having high or moderate effect in each sample were used in the clustering analysis. Clustering of 783 SNPs (Fig. 3a) and 112 structural variants (Fig. 3b) showed that PG2302 from NSB and PG0217 from MSB formed a separate cluster from the other four samples. The difference between the former two and the latter four samples seemed to be caused by the difference in sequencing equipment and by the different timing of the analysis, rather than by unique mutations, suggesting that all NSB and MSB samples had high similarity (Fig. 3).

**Fig. 4:**
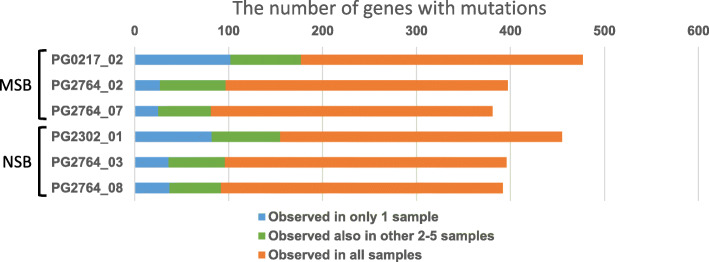
Sharing of SNPs. The number of SNPs in the genic regions shared among three MSB and three NSB samples is shown

Next, we analyzed variants with SNPs observed in one sample, in 2–5 samples, and in all samples (Fig. 4). The variants common to all samples were the most abundant. No considerable difference between NSB and MSB was observed in the average number of variants observed in one sample.

**Fig. 5:**
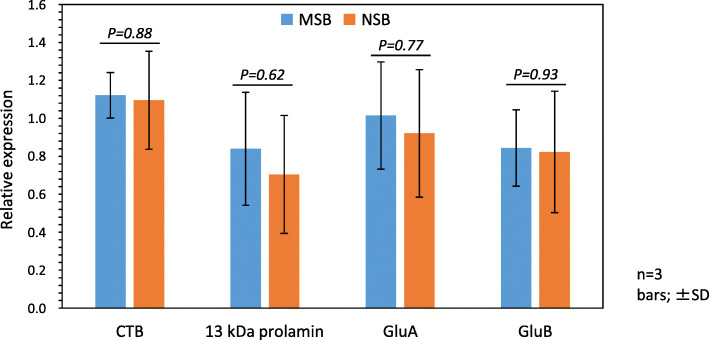
mRNA levels of storage proteins and CTB in MSB and NSB samples. Expression levels were analyzed by qRT-PCR using RNA extracted from developing seeds of 14-DAF plants grown from each three seeds from MSB and NSB. Expression levels are normalized to 17S rRNA and are represented relative to the expression levels of all three seeds in NSB and two seeds in MSB to one seed of MSB. There were no significant differences between MSB and NSB seeds (*n* = 3)

The mRNA levels of CTB and storage proteins (13-kDa prolamin and glutelins A and B) did not differ significantly between NSB and MSB samples (Fig. 5). Taken together, these results further suggest that key genetic characteristics were stably inherited from MSB to NSB.

### Shot-gun MS/MS proteomics analysis in salt-soluble proteins

**Fig. 6:**
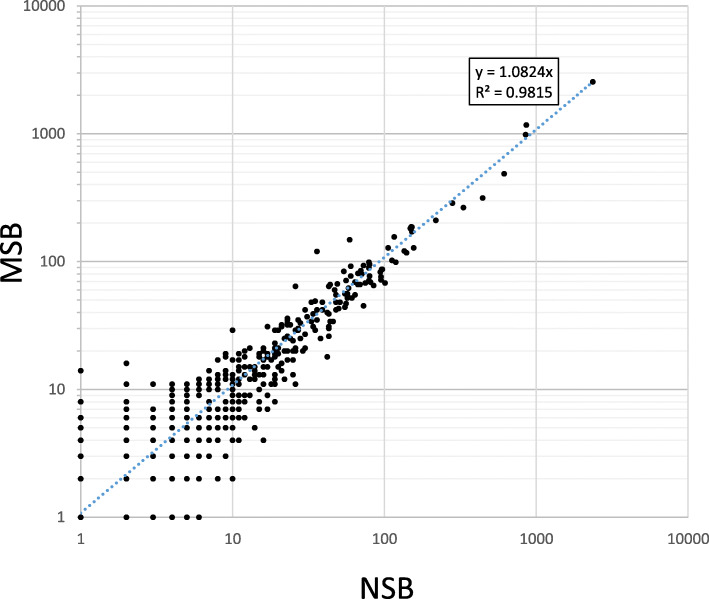
Correlation of PSM values of proteins detected in MSB and NSB samples. Individual peptide spectrum match (PSM) values of proteins detected in MSB and NSB samples by shot-gun MS/MS are plotted

In the salt-soluble protein fractions, we identified 664 proteins in NSB samples and 722 proteins in MSB samples, of which 477 proteins overlapped (Additional file 3: Table S3). We calculated the total number of MS/MS spectra matching peptides for each protein to determine the peptide spectrum match (PSM), which is proportional to protein abundance. We estimated the relative ratio of abundances of the overlapped proteins in NSB and MSB samples from the PSM ratio. A scatter plot of the PSM values showed that the salt-soluble proteins present in the NSB and MSB samples were almost the same (*R*^2^ = 0.982; Fig. 6). The PSM ratio of allergen proteins (63-kDa globulin-like protein, 52-kDa globulin-like protein, 19-kDa globulin, RAG2, RA5, 17-kDa alpha-amylase, and trypsin inhibitors 1 and 2) did not differ considerably between MSB and NSB (Table 2). These findings show that the quality of rice proteins produced by NSB was similar to those of MSB.

**Table 2:** Expression of allergenic proteins in MSB and NSB samples

Description ^a^	Accession	Description^b^	MW (kDa)	Calc. pI	#PSM MSB	#PSM NSB	#PSM NSB/MSB^c^
Putative globulin (63 kDa)	Q75GX9.1	RecName: Full = 63 kDa globulin-like protein; AltName: Allergen = Ory s GLP63; Flags: Precursor	63.4	8.13	2548	2358	0.925
Globulin-like protein (52 kDa)	AAD10375.1	globulin-like protein, partial [*Oryza sativa*]	51.7	10.81	1171	860	0.734
19 kDa globulin	AAA72362.1	unnamed protein product [Oryza sativa Japonica Group]	19.8	6.96	487	614	1.261
RAG2	ACA50505.1	seed allergenic protein RAG2 [Oryza sativa Japonica Group]	17.8	8.03	121	135	1.116
RA5	Q01881.2	RecName: Full = Seed allergenic protein RA5; AltName: Allergen = Ory s aA_TI; Flags: Precursor	17.3	8.03	62	58	0.935
Os07g0216700	XP_015645309.1	17 kDa alpha-amylase/trypsin inhibitor 2 [Oryza sativa Japonica Group]	16.5	7.50	128	106	0.828

^a^Proteins listed as major rice allergenic proteins. ^b^Proteins are annotated in the NCBI database as allergenic proteins. ^c^Expression ratio (NSB/MSB) was calculated by dividing the PSM value of NSB by that of MSB

### Localization of CTB and rice storage proteins in mature seeds by immuno-fluorescence microscopy

**Fig. 7:**
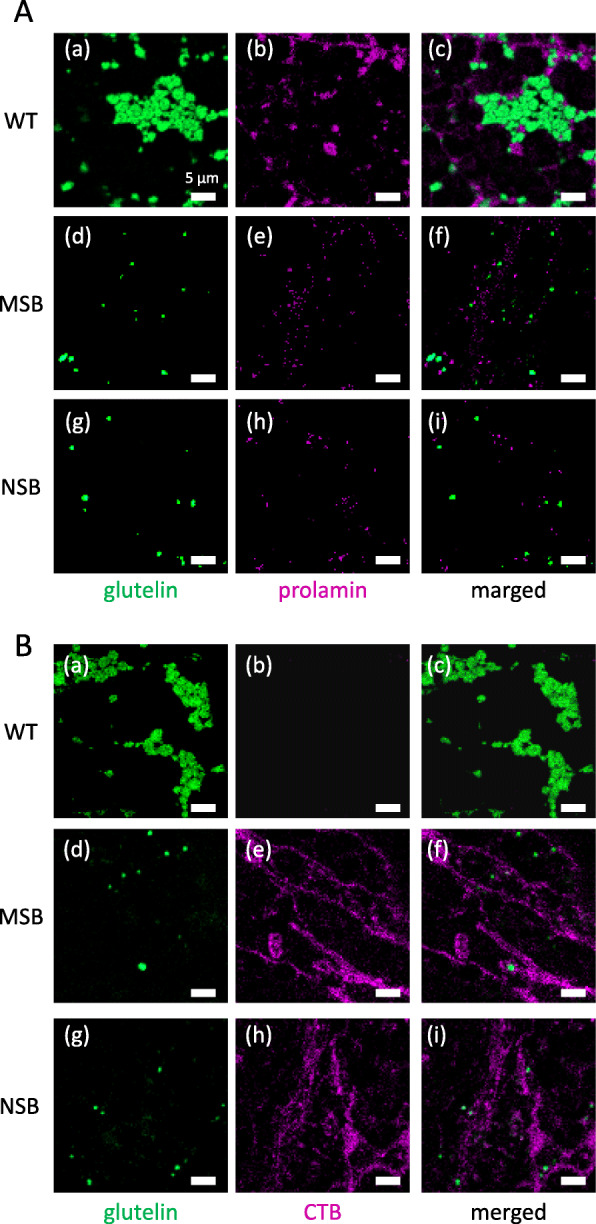
Immuno-fluorescence detection of CTB and storage proteins in mature seeds of MSB and NSB. **a** Double immunostaining with anti-glutelin A antibody and anti-prolamin antibody. **b** Double immunostaining with anti-glutelin A antibody and anti-CTB antibody. Scale bars are 5 μm

Glutelin A and 13-kDa prolamin were found in separate compartments in WT seeds (Fig. 7a (a–c)), consistent with our previous report [14]. The signals of these proteins were much weaker in NSB and MSB than in WT (Fig. 7a (d–f, g–i)). CTB was observed as a network-like structure, which was almost identical in NSB and MSB (Fig. 7b (d–f, g–i)). These results show no difference between MSB and NSB in the level of suppression of rice storage proteins.

## Discussion

Vaccines produced in plants have some advantages over traditional oral vaccines including lower costs, a possibility of rapidly scaling-up production of the vaccine antigen, and no need for purification [1, 2]. Rice seeds can be easily desiccated and are suitable for long-term preservation of a vaccine without need for the cold chain [18]. The requirement for the cold chain is a major burden for vaccination in developing countries because of high costs. Since 2005, we have developed a rice-based cholera vaccine using the MucoRice system and demonstrated that oral MucoRice-CTB induced CT-neutralizing antibodies and protected mice and pigs from challenge with *Vibrio cholerae* or enterotoxigenic *Escherichia coli* [18–20]. We established an MSB of marker-free MucoRice-CTB using line 51A for the production of oral cholera vaccine in 2013 [16]. For clinical trials, we established a prototype of a closed MucoRice hydroponic factory at The Institute of Medical Science, The University of Tokyo, Japan, which was approved as a GMP factory by the Japanese regulatory body, the Pharmaceuticals and Medical Devices Agency (PMDA). The production of MucoRice-CTB was performed a closed hydroponic system for cultivating the transgenic plants to minimize variations in expression and quality during vaccine manufacture. The formulation of MucoRice-CTB was made by polishing and powdering of seed substance and packaged in an aluminum pouch to use clinical trial [17]. We have proceeded to phase I study of MucoRice-CTB and confirmed the safety, tolerability, and immunogenicity of MucoRice-CTB in 2015–2016 in Japan [21] and have performed part of a phase Ib trial in 2019 in the USA (manuscript in preparation). The double-blind, randomized, placebo-controlled, three-cohort, dose-escalation, first-in-human phase I study in Japan showed that MucoRice-CTB induced cross-reactive antigen-specific antibodies against CTB and enterotoxigenic *E. coli* heat-labile enterotoxin in a dose-dependent manner, without inducing serious adverse events [21]. The result of a clinical study in the USA was consistent with that of the phase I study in Japan, suggesting that oral MucoRice-CTB induces neutralizing antibodies against diarrheal toxins regardless of the genetic background.

Because seeds of cereals, including rice, can be preserved in a freezer at about − 20 °C after drying, this method has been used to preserve seeds in a gene bank [22]. However, freezing large amounts of rice seeds may be problematic from an economical and managerial point of view [23]. Thus, dried breeders’ rice seeds intended for large-scale production have been preserved at low temperature without freezing. Because the MucoRice-CTB seed itself becomes the active pharmaceutical ingredient after polishing and formulation of the vaccine, a very large amount of rice seeds is needed to ensure stable vaccine production at the industry level. Therefore, we chose to preserve seeds for a long time under cold conditions with drying. We previously determined the criteria for renewal of a seed bank in consultation with the PMDA in Japan [17]. Although MSBs may be preserved for at least 10 years in the cold after drying, it is not known for how many years we can preserve recombinant rice seeds as MSB. Thus, in this study we produced an NSB from MSB established 5 years earlier to replace MSB in the near future. We produced the NSB in our hydroponic GMP facility and demonstrated that NSB can replace MSB for the production of MucoRice-CTB line 51A by comparative study of these two banks using genomic and proteomic analyses.

We first determined the transgene sequences in NSB and MSB samples. Previously, two transgenes inserted in tandem on chromosome 3 and one transgene inserted on chromosome 12 with the RNAi cassette truncated were sequenced using a primer set that amplifies three fragments from each chromosome [16]. However, the PCR method may have introduced some errors because the amplified fragment was too long. In this study, we changed the PCR enzyme from TaKaRa LA Taq Hot Start Version (TaKaRa Bio, Inc.) to KOD FX Taq (Toyobo Biochemical Co.) and used primer sets that anneal to positions where amplification is efficient (Fig. 2). We revised one position in the transgene sequence on chromosome 3 and five positions on chromosome 12 in MSB and found that the transgene sequences in NSB were identical to those of MSB. The transgenes were inserted into intergenic regions on both chromosomes [16], so it is unlikely that their insertion would affect transcription.

Regarding genome analyses, NSB (PG2302) and MSB (PG0217) had larger total read numbers than the other four samples (Table 1) because they were sequenced on different next-generation sequencers. However, no considerable differences between the mapped read numbers and mapping rate were found among the six samples. The depth average was over 30 for all samples, the mapped sequences were reliable [24], and the NSB (PG2302) and MSB (PG0217) depth averages were larger than those of the other samples because of the higher numbers of total reads.

We have not performed genome-wide gene expression profiling for the seed bank, but we have compared the genic regions of the chromosomes in NSB and MSB samples by examining whole-genome sequence datasets. We focused on SNPs and structural variants (e.g., translocations, deletions, and inversions) in the genic regions that may affect the phenotypes of MSB and NSB. In clustering analysis based on SNPs and structural variants that were evaluated to have a high or moderate effect by the criteria listed in Table S2, mutations in NSB (PG2302) and MSB (PG0217) were classified into different branches from those of four other samples, which we explained by the differences in the equipment used for sequencing (Fig. 3). These results indicate that there are no obvious differences in SNPs and structural variants in the genic regions between NSB and MSB samples. Although whole-genome sequence analyses of WT and GM rice with T-DNA insertions have been published [25, 26], this is the first report that NSB obtained by self-pollination was almost identical to MSB at the genomic level. In addition, we found that 70% of the variants in the genic regions were shared in all six samples, and there were very few mutations observed in one sample only in NSB and MSB (Fig. 4). We concluded that the renewal of MSB did not affect transgenes or genic genes. Thus, NSB can be used instead of MSB, leading to the creation of a sustainable seed bank system for MucoRice-CTB51A.

Using shot-gun MS/MS, we also identified salt-extracted proteins from MSB and NSB but did not perform a comprehensive protein expression profiling (such as clustering). By proteomics analysis, we have shown that the levels of 63-kDa globulin, 52-kDa globulin-like protein, and glyoxalase, the major allergen protein candidates, were the same in MucoRice-CTB and WT, whereas the levels of 19-kDa globulin, RAG2, RAG5, and 17-kDa alpha-amylase/trypsin inhibitor were low [15]. In the present study, the levels of these allergen proteins were similar in NSB and MSB (Table 2). Using the PSM values of salt-soluble proteins other than the major allergen proteins, we showed that protein levels in NSB and MSB samples were highly correlated in a scatter plot (Fig. 6).

We also compared the mRNA levels of glutelin A, glutelin B, 13-kDa prolamin, and CTB in NSB and MSB samples (Fig. 5). In MucoRice-CTB51A, mRNA levels of the storage proteins glutelin A and 13-kDa prolamin are lower than in WT because of RNAi suppression [12]. NSB and MSB had almost the same mRNA levels of glutelin A, glutelin B, 13-kDa prolamin, and CTB. To increase the accumulation of CTB in rice seeds, RNAi sequences specific to genes for 13-kDa prolamin and glutelin A were introduced into MucoRice-CTB to suppress their expression [14]. We performed immuno-fluorescence microscopy on mature seeds (Fig. 7) and, as expected, found very low signals of prolamin and glutelin A in both NSB and MSB samples in comparison with WT rice. In NSB and MSB samples, CTB formed a network-like structure, which may represent a developing protein storage organelle. Foreign proteins produced in endosperm tissue have been found to be localized in a unique structure distinct from normal rice protein storage organelles [27–29]. Production of foreign proteins may lead to dysfunction of binding protein (BiP), which is a chaperone controlling protein folding in the endoplasmic reticulum, resulting in unique protein distribution or formation of a unique structure (e.g., network structure) [30]. In a previous study, the observation of CTB in immature seeds of MSB harvested 14 days after flowering (DAF) showed that CTB was mainly localized in the cytoplasm and protein body (PB)-like structures near the cell wall [14]. In the present study, we examined mature seeds of MSB and NSB. Saito et al. [31, 32] showed that endosperm cells are filled with starch granules, PB-Is, and PB-IIs in the appearance of mature seeds, and these storage organelles continue to develop after 14 DAF. Therefore, PB-like structures in 14-DAF seeds of MSB and NSB may change during seed maturation and drying. Although NSB and MSB have unique protein distribution and/or structure compared with WT, the quality of CTB and rice proteins produced in them are consistent, as judged from our current data.

Tekoah et al. [10] have developed a plant cell culture process for the production of taliglucerase alfa and established a master cell bank for a future production line and a working cell bank for continuous manufacturing from the initial cell line. This cell line can be stored for a long time in a deep freezer, similar to bacterial and mammalian cell cultures [10]. Ma et al. [11] defined the T6 generation as a working seed bank (WSB) derived from the T5 generation (MSB) of tobacco plants expressing the monoclonal antibody P2G12. Ma et al. carried out Southern blot analysis of three individual plants from T5–T7 generations using probes specific for the heavy and light antibody chain genes and estimated that this line contained two copies of the heavy chain gene and six copies of the light chain gene. They confirmed the identity of the transgenes and immediately adjacent sequences on the chromosomes of T5 and T6 from three individuals per generation by sequencing analysis [11].

## Conclusion

In this study, we generated NSB from MSB. To demonstrate the consistency between the two seed banks of MucoRice-CTB51a, we compared the MSB and NSB samples in terms of the sequences of the inserted transgenes and the genic regions, the transcription of the integrated transgene, the levels of salt-soluble proteins, and protein location in the endosperm tissue of mature rice seeds. All analyses revealed that NSB was of the same quality as MSB, indicating that MSB can be renewed. Plant seeds are an attractive production system for pharmaceutical proteins, but there have been few studies on the establishment and renewal of seed banks. We expect the approach used in this study to become an important evaluation method to advance the development of vaccines stably produced in recombinant plants to clinical applications. We propose that this is a model case study for the administration and renewal of transgenic rice seed banks.

## Methods

### Next seed bank of MucoRice-CTB line 51A

The selection marker–free MucoRice-CTB line 51A (T6 generation) [16] was cultivated in a fully closed GMP plant production facility (Asahikogyosha Co., Ltd., Tokyo, Japan) built at The Institute of Medical Science, The University of Tokyo, as described in [17]. The CTB protein was quantified in MSB and NSB by SDS–PAGE using a calibrated GS-900 densitometer (Bio-Rad Laboratories, Hercules, CA, USA) as described previously [12, 17].

### PCR analysis and confirmation of transgenes on chromosomes 3 and 12

Each of the three seeds (six in total) selected randomly from MSB or NSB was hydroponically cultivated with nutrient solution (OAT House fertilizers 1, 2, and 5; OAT Agrio Co., Ltd., Tokyo, Japan) in a growth chamber under a 12-h day (27 °C)/12-h night (22 °C) cycle. Genomic DNA was isolated from young leaves of each seedling 3 weeks after germination by using a Nucleon PhytoPure kit (GE Healthcare, Madison, WI, USA). The primer sets for PCR are listed in Additional file 1: Table S1. PCR was conducted by using KOD FX Taq (Toyobo Biochemical Co., Osaka, Japan) and a GeneAmp PCR System 2720 (Applied Biosystems, Carlsbad, CA, USA) under the following conditions: 2 min at 94 °C; and 30 cycles of 10 s denaturation at 98 °C, 5 min annealing and extension at 68 °C. The PCR products were separated by electrophoresis on a 1.0% (w/v) agarose gel, excised from the gel, and cloned into the pTA2 vector. The cloned PCR products were sequenced by Fasmac Co., Ltd. (Kanagawa, Japan) and the sequences were analyzed with Genetyx software (Genetyx, Tokyo, Japan).

### Whole-genome re-sequencing

Data from the previously described whole-genome re-sequencing of MSB_1 were used [26]; these data were obtained with an Illumina HiSeq2000 platform (Illumina, San Diego, CA, USA). Genomic DNA extraction and sequencing of NSB_1 was performed in 2018; for MSB_2, 3 and NSB_2, 3, we used two genomic DNA samples (1.0 μg, each) out of the three described in the previous subsection [26]. DNA of the NSB_1 sample was sequenced with a HiSeq2500 platform (Illumina). DNA of the MSB_2, MSB_3, NSB_2, and NSB_3 samples was sequenced with an Illumina NovaSeq6000 platform (Illumina). Paired-end read sequences (100 bp per read; Sanger FASTQ format) from both sides of each fragment were obtained with Casava software (ver. 1.13.48; Illumina). This analysis was performed using the Genedata Profiler Genome software (Genedata). Reads were mapped to genome sequences using BWA-MEM, and then duplicate reads were removed.

### SNP and InDel detection

SNPs and short InDels between the mapped read data and the reference genome were called with SAMtools as described in detail in [26]. All variants from the six samples were listed according to SAMtools. Using SNPEff software and publicly available rice data sets, we predicted the effects of variants on protein function and categorized all of the sample-specific variants into effect types, which we then grouped into four larger categories (HIGH, MODERATE, LOW, or MODIFIER) on the basis of the assumed severity of each effect (Additional file 1: Table S2).

### Clustering analysis of variants

The number of mutations was counted for each gene, which was considered “mutated” if it had at least one HIGH or MODERATE mutation. Genes with “NA” in any sample were excluded (783 genes). Hierarchical clustering was performed for each gene and sample using Cluster 3 (centroid linkage with Pearson’s correlation coefficients) [33]. For clustering by paired-end sequences, any detected mutated locus annotated as HIGH or MODERATE was clustered (112 loci) as above. Loci or genes with no mutations in any samples were excluded.

### Quantitative real-time PCR

Each of the three seeds (six in total) selected randomly from MSB or NSB was hydroponically cultivated with nutrient solution (OAT House fertilizers 1, 2, and 5) in a growth chamber under a 12-h day (27 °C)/12-h night (22 °C) cycle. Six RNA samples were extracted from developing seeds harvested at 14 DAF with a RNeasy Plant Mini Kit (Qiagen, Hilden, Germany). Samples were treated with DNase (TaKaRa), and cDNA was synthesized from total RNA (0.5 μg) with a PrimeScript RT Reagent Kit with gDNA Eraser (TaKaRa). cDNA (20 μL) was diluted 1:50 with distilled water. Quantitative real-time PCR (qRT-PCR) was performed in triplicate in a total volume of 20 μL containing 0.5 μM each primer and Fast SYBR Green Master Mix (Applied Biosystems) on a StepOnePlus Real-Time PCR System (Applied Biosystems) (95 °C for 20 s; 40 cycles of 95 °C for 3 s, 60 °C for 30 s; followed by 95 °C for 15 s and 60 °C for 1 min). The primer sets are shown in Additional file 1: Table S4. The expression levels were normalized to 17S rRNA levels. The results were compared by using unpaired two-tailed Student’s *t*-tests. All statistical analyses were done in Prism 7 (GraphPad Software, CA, US).

### Shot-gun MS/MS

Mature brown seeds (about 0.5 g each) from MSB or NSB were collected randomly and pulverized with a Multi Beads Shocker (Yasui Kikai Corp., Osaka, Japan). The salt-soluble proteins were extracted from 0.2 g of the rice fine powder with 3 mL of 1 M NaCl by rotating for 3 h at 4 °C on a rotator (Taitec Corp., Saitama, Japan), and centrifuged at 20,400×*g* for 10 min at 4 °C. The supernatant was filtered through a 0.45 μm syringe filter and stored in aliquots at − 80 °C until use. Shot-gun proteomic analyses of the peptide mixtures were performed by using a linear ion trap–orbitrap mass spectrometer (LTQ-Orbitrap Velos, Thermo Fisher Scientific) coupled with a nanoflow LC system (Dina-2A, KYA Technologies, Tokyo, Japan) as described in [15]. Proteins were identified by searching the MS and MS/MS data against the National Center for Biotechnology Information (NCBI) non-redundant rice protein database by using Mascot (Matrix Science). We also conducted decoy database searching by using Mascot and applied a filter to satisfy a false-positive rate of less than 1%.

### Immuno-fluorescence microscopy

Mature seeds were embedded by a modified method of Saito et al. [32]. The seeds were cut into 0.5–1.0 mm sections and fixed for 3 h in 4% (w/v) paraformaldehyde in 0.1 M sodium phosphate buffer (pH 7.2) at room temperature. Then, the sections for the immune-fluorescence microscopy were prepared as described in detail in [14]. For CTB and glutelin double staining, the sections were incubated with avidin and biotin solutions (Vector Laboratories) (15 min each), followed by blocking with 5% donkey serum in PBS for 30 min at room temperature, and incubated with rabbit anti-CTB antibody (10 μg/mL) or mouse anti-glutelin antibody (5 μg/mL) in 1% BSA in PBS for 1 h. The sections were washed with PBS and incubated with biotin-SP-conjugated donkey anti-rabbit IgG (Jackson) (1:200) in 1% BSA in PBS containing 10% normal calf serum for 30 min. The sections were washed with PBS, incubated with Streptavidin-Alexa Fluor 467 (Invitrogen) (1:200) and DyLight488-conjugated anti-mouse IgG antibody (1:200) in 1% BSA in PBS for 1 h, and washed with PBS. For prolamin and glutelin double staining, the sections were blocked with 5% donkey serum in PBS for 30 min and incubated with rabbit anti-13 kDa-prolamin antibody (1:1000) or mouse anti-glutelin antibody (5 μg/mL) for 1 h. The sections were washed with PBS and incubated with Cy5-conjugated anti-rabbit IgG antibody (1:200) or DyLight488-conjugated anti-mouse IgG antibody (1:200) in 1% BSA in PBS for 1 h, followed by washes with PBS. Images were captured with a confocal laser scanning microscope (LSM 800 Axio Observer, Carl Zeiss, Germany).

## Supplementary Information

**Additional file 1: Table S1**. Primer sets for amplification of the transgene; **Table S2**. Summary annotation impact and annotation type; **Table S4**. Primer sets for quantitative real-time PCR

**Additional file 2: Figure S1**. Sequences of transgene regions of NSB on chromosomes 3 and 12

**Additional file 3: Table S3.** List of salt-soluble proteins of MucoRice-CTB identified in both MSB and NSB by shot-gun MS/MS analysis

## Data Availability

The data sets supporting the results of whole-genome re-sequencing of this article are available in the DNA DataBank of Japan (DDBJ) Sequenced Read Archive under the accession number DRX011151. The data sets are also available in NCBI Entrez: https://www.ncbi.nlm.nih.gov/sra/?term=DRA011151

## Abbreviations

CTB: Cholera toxin B subunit
GMP: Good manufacturing practices
MSB: Master seed bank
NSB: Next seed bank
PBS: Phosphate-buffered saline
PMDA: Pharmaceuticals and Medical Devices Agency
PCR: Polymerase chain reaction
RNAi: RNA interference
SNP: Single nucleotide polymorphism
T-DNA: Transfer DNA
WSB: Working seed bank
WT: Wild type
