# Supplementary material for: Comparative whole-genome and proteomics analyses of the next seed bank and the original master seed bank of MucoRice-CTB 51A line, a rice-based oral cholera vaccine
Source: BMC Genomics. 2021 Jan 19;22:59. doi: 10.1186/s12864-020-07355-7 (PMC7814724; doi:10.1186/s12864-020-07355-7)
Supplement: Supplementary file 1 — Additional file 1: Table S1. Primer sets for amplification of the transgene; Table S2. Summary annotation impact and annotation type; Table S4. Primer sets for quantitative real-time PCR [file 12864_2020_7355_MOESM1_ESM.pdf]

Table S1. Primer sets for amplification of the transgene

3 chromosome	
ch3#51A-F	5'– GGCCAGCTGCACAACCCTCA –3'
ch3#51A-R	5'–ACAGCAGGGAAGTGCCTGGA –3'
ch3#51A-LongPCR-F	5'–GGAGCTTGAGCTTTAGACAACTTAATAACAC–3'
ch3#51A-LongPCR-R	5'–AAGTTGTCTAAAGCTCAAGCTCC–3'
RAPint3-R	5'–ACATGAGATATGCCACCAGC–3'
RAPint4-F	5'–ATGTTGTGACTGTCTGCCTC–3'
12 chromosome	
ch12#51A-F	5'–GGAGTCAAGACTGCGACACA–3'
ch12#51A-R4	5'– AGCTGGTCCTCTTCAGTTGC–3'
CTB-F2	5'–ACACCTCAACAGATTACTGATTTG–3'
CTB-R	5'–TCAATTTGCCATACTAATTGCG–3'

Table S2. Summary annotation impact and annotation type

Annotation Impact	Annotation
HIGH	chromosome_number_variation
	exon_loss_variant
	frameshift_variant
	rare_amino_acid_variant
	splice_donor_variant
	start_lost
	stop_gained
	stop_lost
	transcript_ablation
MODERATE	3_prime_UTR_truncation + exon_loss
	5_prime_UTR_truncation + exon_loss_variant
	cording_sequence_variant
	disruptive_inframe_deletion
	disruptive_inframe_insurtion
	missense_variant
	regulatory_region_ablation
	splice_region_variant
	TFBS_ablation
LOW	5_prime_UTR_premature start_codon_gain_variant
	initiator_codon_variant
	splice_region_variant
	start_retained
	stop_retained_variant
	synonymous_variant
MODIFIER	3_prime_UTR_variant
	5_prime_UTR_variant
	coding_sequence_variant
	conserved_intergenic_variant
	conserved_intron_variant
	downstream_gene_variant
	exon_varaint
	feature_elongation

feature_truncation
gene_varint
intergenic_region
intron_variant
mature_miRNA_variant
miRNA
NMD_transcript_variant
non_coding_exon_varaint
non_coding_transcript_variant
regulatory_region_amplification
regulatory_region_variant
TF_binding_site_variant
TFBS_amplification
transcript_amplification
transcript_variant
upstream_gene_variant

SNPs and structural variants on cording region may affect protein function were categorized into 48 types. These types were further grouped into HIGH, MODERATE, LOW, and MODIFIER according to potential severity. The assignment criteria were pre-defined in the annotation program (SNPEff).

Table S4. Primer sets for Quantitative real-time PCR

CTB-F	5’–AGAGAGATGGCTATCATTACTTTCAAGA–3’
CTB-R	5’–TGAATCTATATGTTGACTGCCTGGTACT–3’
13-kDa prolamin (RM1)-F	5’–CAGGCTGGTAGCGCAACA–3’
13-kDa prolamin (RM1)-R	5’–ACAATCGCCTGAACGCTACT–3’
glutelin A (Glu A)-F	5’–ACAAAGAGAAGGATGTGCTTAC–3’
glutelin A (Glu A)-R	5’–ATTCTTTATCCGCATTGCCAAC–3’
glutelin B (Glu B)–F	5’–CAAGACAAACGCTAACGCCTTC–3’
glutelin B (Glu B)–R	5’–TCGATAATCCTGGGTAGTATTG–3’
17S rRNA-F	5’–TTCCGGTCCTATTGTGTTGG–3’
17S rRNA-R	5’–ATGCTTTCGCAGTTGTTCGT–3’
