# Supplementary material for: Comparative whole-genome and proteomics analyses of the next seed bank and the original master seed bank of MucoRice-CTB 51A line, a rice-based oral cholera vaccine
Source: BMC Genomics. 2021 Jan 19;22:59. doi: 10.1186/s12864-020-07355-7 (PMC7814724; doi:10.1186/s12864-020-07355-7)
Supplement: Supplementary file 2 — Additional file 2: Figure S1. Sequences of transgene regions of NSB on chromosomes 3 and 12 [file 12864_2020_7355_MOESM2_ESM.pdf]

[The sequence of transgene on Chromosome 3]

```
1    ATATATTGTG GTGTAAACAA ATTGACGCTT AGACAACTTA ATAACACATT GCGGACGTTT 60
61   TTAATGTACT GAATTAACGC CGAATTGCTC TAGCATTCGC CATTCAGGCT GCGCAACTGT 120
121  TGGGAAGGGC GATCGGTGCG GGCCTCTTCG CTATTACGCC AGCTGGCGAA AGGGGGATGT 180
181  GCTGCAAGGC GATTAAGTTG GGTAACGCCA GGGTTTTCCC AGTCACGACG TTGTAAAACG 240
241  ACGGCCAGTG CCAAGCTGGC GATCGCTTTG GCGCGCCAAG CTTTTTGTCT AGTTGGCAGC 300
301  CTAATTAATT CTATGGAAAC CAGGTGACAT GGAGGGTTGG GGACATGGTG GAAAAAACCG 360
361  GAACGGGCCG ACAGTTCAAC CGGAAAAAAC CAGAACCCGT TCAGTTCAAA AGAAAGACCG 420
421  GACATGCATA TGACCCGCTT TGAACCGGCA GAACCGGTCG GTTTTTCTAT GAACCGGTCA 480
481  TTAAACCGTC CCCGGTTAGA CCGAACAAGC CACAATAATC TTGAAATGGG CCTTGATGTG 540
541  GCCCAATTGG TCTGCCTAGA GCGTTTTGGT TGGCAAAAAT CAATCTCCTA TTCTCGGCAC 600
601  GTGTGATATA CAATGGTAAG TGAGATATAC AATTCTCGGC ACGGCTACAT TACAAGGTGT 660
661  CGCATTGTGT CAATGTTTGG TTAATTTGCT AGATTCACAT AATACATGCC AGGAAGTTCA 720
721  GAACAATGTG TTGCCTTTCA CCGGAAAACT TTGTTGGAGC AAATGCCTTC TTCTTTTTTG 780
781  CTTCTGCTTC TTGAGTCCAT GTGGAGGAAG CAGTAGATAG CTGATGATAT CAGGATTCCT 840
841  TCTGTGTCTG TGTAGGTGTA GCAACACCAC TATAATTTTT ATTTAGCAAC ACAATATCAA 900
901  TTTGGTCTAT AAAAGTATGA ATTAAATCAA TCCCCAACCA CAATTAGAGT AAGTTGGTGA 960
961  GTTATTGTAA AGCTCTGCAA AGTTAATTTA AAAGTTATTG CATTAACTTA TTTCGTATCA 1020
1021 CAAACAAGTT TTCACAAGAG TATTAATGGA ACAATGAAAA CCATTGAACA TACTATAATT 1080
1081 TTTTTTCTTA CTGAAATTAT ATAATTCAAA GAGCATAAAC CCACACAGTC GTAAAGTTCC 1140
1141 ACGTGTAGTG CATTATCAAA ATAATAGCTT ACAAAACATA ACAAACTTAG TTTCAAAAGT 1200
1201 TGCAATCCTT ATCACATTGA CACATAAAGT GAGCGATGAG TCATGTCATT ATTTTTTTGC 1260
1261 TCACCATCAT GTATATATGA TGGGCATAAA AGTTACTTTG ATGATGATAT CAAAGAACAT 1320
1321 TTTTAGGTGC ACCTAACAGA ATATCCAAAT AATATGACTC ACTTAGATCC TAATATAGCA 1380
1381 TCAAGCAAAA CTAACACTCT AAAGCAACCG ATAGGGAAAC ATCTATAAAT AGACAAGCAT 1440
1441 AATGAAAACC CTCCTCATCC TTCACACAAT TCAAACATTA TAGTTGAAGC ATAGTAGTAG 1500
1501 AATCCTACAA AATCTAGTAT TGTAGAATCA GCAATGGCAG CATACACCAG CAAGATCTTT 1560
1561 GCCCTGTTTG CCTTAATTGC TCTTTCTGCA AGTGCCACTA CTGCATCTAG AACACCTCAA 1620
1621 CAGATTACTG ATTTGTGTGC AGAATACCAC AACACACAGA TCCACACCCT CAATGATAAG 1680
1681 ATTTTCTCTT ATACAGAATC TCTAGCTGGA AAGAGAGAGA TGGCTATCAT TACTTTCAAG 1740
1741 AATGGTGCAA CTTTCCAAGT AGAAGTACCA GGCAGTCAAC ATATAGATTC ACAAAAGAAG 1800
1801 GCAATTGAAA GGATGAAGGA TACCCTGAGG ATTGCATATC TTACTGAAGC TAAAGTTGAA 1860
1861 AAGCTATGTG TATGGAATAA CAAGACTCCT CATGCAATTG CCGCAATTAG TATGGCAAAT 1920
1921 TGAGAGCTCA TTGTAATAGT ATAATGGTTC AAATGTTAAA AATAAAGTCA TGCATCATCA 1980
1981 TGCGTGACAG TTGAAACTTG ATGTCATATA AATCTAAATA AAATCACCTA TTTAAATAGC 2040
2041 ATTCATGTAT GAGGTTGCAT TATCATAGCT AATTACCATC ACAAAGAACT TTACAATTAC 2100
```

2101 TATGTGCATG CATTTGATCC TAAGCTACTT TGGCTATTAG ATACAAATGG AGTGTATTAA 2160

2161 GCAAGTCCAA CTTTTCATTC TAATAGGAAC AAACTTGAAG CACATATGTC AATTACTATA 2220

2221 TCCCCAACAA TTGATACACT ATAGCAGTGG TTGCAATGGC AGCTGCAGGA ATTGCTAGTG 2280

2281 AACCCAACAG CCCATATAAC TGAATTGAAG CCATGTCAGA CATCCGTAAT GAACAACAAA 2340

2341 TAATTCCAAA TAGTGCATAA CCAATAAATA CTTAAAGATT TGTCTTTCAA CACTAATGTT 2400

2401 TGCATTTCAT GCAATTGTAT TGCTCTGGAA GCACCTTTTA ACAGGTTGTA TCATCACATG 2460

2461 CATTACATTG GAGGATAAGG ATATAAGAGA TCATATATAG AATAGAGGAT ATGGTGTTTT 2520

2521 AGAAAAAGAA GATGAAGAAT ATACATTGTG AAACAGTGCA TGATATAGGC ACATCACTTA 2580

2581 ATACATTAAA AGCACTGCCA GAAATCTATG TTGGTGAACA AATGCTCACT GGTTGTCTCA 2640

2641 CTTATATCAC CATTCACCAA GTTCACCGTA TGTAACAAGC ACTTGGCCCC ATGAATTCAC 2700

2701 GCGCCAAGCT TGTAGTGAAC GGTCTGGACA GAGATTCGTC GCGTCGTCAG CGGAAGGAGG 2760

2761 TGCTTGAATC CCTGATGCTG CCGGTTCTCT GTGTGTTTTT GGCTTGTGAG ATGGGGTTTA 2820

2821 GTCAGTGCAA TTTCAGTGGA TTTTGCTCAA CTCGGTTGGA TTGGCACGAG ATTGGTTCCG 2880

2881 CGTGTGACCG TTTGTTGGAG AACATGAAAT CGCGTTGCTC GTTCTGGTTT GGATGCTCTG 2940

2941 TTTTCTGAAC TAGGTTGCTC GAATTGAATC TGATTGGTTT ATGTCTACCT CCGAGTATCA 3000

3001 AGTAAGTTCA GATGAGCGGT TAGCTCGTTT TCAGAGCTAG ACGGGATCAC GATCTGGGAA 3060

3061 TACAATTTTT ATGAGGCAAA TGCAGCAAAT ATAAAGTGAT CTAACTAATG CCACTTGATA 3120

3121 TTATGATGAA CATGTTGTTG TGAAACTGGA AAATGCATGC CAAATTGCCA ACCATATACA 3180

3181 TCTCATTAGT AGAGTAACTT CGTGACAGTG AAAGAAAACT TCATTCTAGC TGAGGGGAGA 3240

3241 TCACATTGAA GGTATTCGTG ATATAATATG TTAGCAAGAT AAGTAGCTAG GATGGTTTAT 3300

3301 TCATAGCACA TTGGTACCTG GCAGCATGTC AGATAGATAT TTTCTATTTC AGCAATATAT 3360

3361 TGATCTTACC TTGTGCAACA GTTTTCTCCA TCCAAGTTCA GTGAAAATCA TTTGTCCTTG 3420

3421 AACTTATCTA CTCAAGCGAT GAACAATACT CATACACATG AGCATATGAA TCAGTTCTAT 3480

3481 CTTCTATGAA GCCTTAACCC TAGATTTATC ACAATCTAGT CATCTAGTCC GGCAGACCAC 3540

3541 ATTGAACTCC ATTGCAATTT GTGAGTTGTT GTATGGCTTC GGAATGGGGT CGAATCTTTC 3600

3601 TTCAATCCAG GATGGAAATT TTGAGATCTA TACATCACGT TTGGTACATC TCTACTACTC 3660

3661 TAGTACTAAA AACATGAAGA TTCTGAACAT GGACATTGCA TAGTTCTCCA TCCCAATTCA 3720

3721 CCCTGCAGTG ATCGCTGCAC TGGATAATTA TAATATCAGT TAAAATTGAA AATAATGCAA 3780

3781 CTTCATACTT GCATGGTGTC AGTAGTGCCT GCCTAAGAAA TGTGTCTTGT CATAATATGA 3840

3841 TTACATGAAA TATGTTTACT TCCTCGTTTC TCTTTATTTG TAAGATAAAG AACTAGATAT 3900

3901 GTGGAAAGTA GGATAGCAAA GAGTATGGCC AAACTCTAAT CTTTGCTTTA TTTTTTGGGA 3960

3961 TGGACCCAAA ATTTGTTTCT CCTTTACTTC TTTCCCTTTA CAACAATGTT CTTTACTTCC 4020

4021 AATTCTTATT AACAAAACTC CAAATACATG CCAAACTGCA TATGTATGTA TGCTATTAAG 4080

4081 GCACATTTAC AAAGCTCCAA GTTTACCTAC TCAATCATTC ACATATGGCG ATGACTCAAA 4140

4141 CTCTTAATTG TTATCTGTGT AAGCTGTGAC TTGTGTAACA CATTCTACAA GTCCCATACC 4200

4201 AATTCTGTTC ACAAAAGTTT CTTTGTCCAG CTCATAATTT ACAAAACTGC AAAATGCCAA 4260

4261 AGCAATCTGG CACAACCTTA TCATCATATT TTCTTTCCAC GCATTAAAGC ACTGGCAGAA 4320
4321 TTATCTTTGT GTAGATATTC CAAAAGTATT GGTTGAATAA ATGTCCAAAT AAATTCCATG 4380
4381 CCTCATGATT TCCAGCTTAT GTGGCCTCCA CTAGGTGGTT TTGCAAAGGC CAAACTCTTT 4440
4441 CCTGGCTTAC ACAGCTACCA GCATGTATAA ATAGGCCCCT AGGCAACCAT TATTCCATCA 4500
4501 TCCTCAACAA TATTGTCTAC ACCATCTGGA ATCTTGTTTA ACACTAGAAT TGTAGAATCA 4560
4561 GCATCTAGAA GGGCAACCGG GGAAGGTTAA ACCCATAGAA CCTCTCCCTT GGATGATGTA 4620
4621 GACCACGCCA GGAATATTTG TGTATCGAGG TACCAAAAGG CCTTGAGGCT GAATGACACG 4680
4681 TCGGATCACA AAAGTACCCG TGCACTGGAA TAATTCATTC TTCTCATCGA AGTACTCAGT 4740
4741 CACCCCAGCT TCTGACCTCA CTTTCCGAAG TGGTTCAAAT GCTTGTAGTC TATCAAAACT 4800
4801 AGAGCATGCA ACAATAGCAA GGAGAGCAAA TACGAAAATG ATCTTCATAC TAGACCCAGA 4860
4861 TCTCAGAAGA ATGGTCCGTG CCTTAAAACT TTCCCCAACC GTGCTAGTTT ATGTTGTGAC 4920
4921 TGTCTGCCTC TCTCAGTTTA CTTGGATGCA TTGACAACAT CCTTTTTTGC TATTACTCGT 4980
4981 ATTTGCTCTA TAGCTGGTGG CATATCTCAT GTTGAAATTT GCCCTTTTAA TCCAAAATTG 5040
5041 GATGTAATTG AAAGAATCCT ACGTGGTAGT TATTTGGATT TTGGTGTGAA AAAAAATAGC 5100
5101 CTTGTTAGAA GAAGCAAAAT TGGATTTAGT TAAAAGGATA CTAGATGGTG TTATTTGGAT 5160
5161 TTTGGTGCAA ATCAAATTAG GAGGTTGGTT TTATTCAAGT TAAAGTTTGT TTTAAAAAAA 5220
5221 TTCTCCTAAA AAGATAGATA CTAGATTTGC ATATATGCAT TGAAAATTAC ATCTTCGCTT 5280
5281 GGCGGTTATA CTTTTAGTCC CTCTAAATTG TTCAATCATT TATGATGAAA AGGAAAATCA 5340
5341 TTTTATATCA CAAAGTATTT ATGATGAAAG GGGAAAAATA TTCTGCATGG GTTTGAACAA 5400
5401 AATACGTGGA TTGGTGTAGC CTTAACATAC TTGAAAAGGG TATGATGTTG ATGTAGTGCC 5460
5461 CACATGGTGT CGCTTGACAT TAAAACGATA TGCAGTCAGG ATTGAGGAAC ATTGCTGACA 5520
5521 ATTTACTATC GCTGTCTGTG TTGACCACAA TAATTCAGAT GTACCATCCT ATCTTCTAAC 5580
5581 TAGAAAGATG CATGGAAGTT TCTTACATTA TTTCCAGCAC TTGAAATTTT AGTGAAATAT 5640
5641 CATTAAAACA TAACCACTTA CTTTGCTGTG ATATGAAATA AATGTTTTAT TTCTTGGAAA 5700
5701 GTGGTATATT CATATATTCT TACAGTAAAT TTATTGATTT TCTTTTCATT TATTTCTAAA 5760
5761 TTTTAACCAC CCTTTTGGTA GCTTAAGGAA AATTGTATGT TTGACAGTCC TGTTTTCTGT 5820
5821 TGTTTCATCC CTCCAGGAAA ACCAGCTACT AGTATGAAGA TCATTTTCGT ATTTGCTCTC 5880
5881 CTTGCTATTG TTGCATGCTC TAGTTTTGAT AGACTACAAG CATTTGAACC ACTTCGGAAA 5940
5941 GTGAGGTCAG AAGCTGGGGT GACTGAGTAC TTCGATGAGA AGAATGAATT ATTCCAGTGC 6000
6001 ACGGGTACTT TTGTGATCCG ACGTGTCATT CAGCCTCAAG GCCTTTTGGT ACCTCGATAC 6060
6061 ACAAATATTC CTGGCGTGGT CTACATCATC CAAGGGAGAG GTTCTATGGG TTTAACCTTC 6120
6121 CCCGGTTGCC CTTCTAGTGG ATCCCCGGGT ACCGAGCTCA AACGTTGGTT ACATGTACTC 6180
6181 TAGTAATAAG GTGTTGCATA CTATCGTGTG CAAACACTAG AAATAAGAAC CATTGAATAA 6240
6241 AATATCAATC ATTTTCAGAC TTGCAAATAT TGGGTATTTG GATTTCTGTC CCATGTCCCT 6300
6301 CTTGAAAGCC ATGCTGTACA TGTTGGAGTT CCCCCTTGGA CCCAACCTAC TCCATGCTCC 6360
6361 CATGTTGATC TTAAATTCCC TGTTCCCCCA GAGCATGTAA ATTTTCTTAT GCTAATCAGA 6420

6421 GCAAGCTCGA TGTCTCATTA ACATATCCCT ATTTGATCAA TGTTGTGGTT GTTTCAAAAT 6480
6481 GTATTGGTGA TAAGTGGTAA CAGTGTACTT CTGGCATAAT GGATAATAAG TGGCGGAGGT 6540
6541 TTGTGAAGAT AAGAGTTGGA AACAGAGCTG CTGTGTATGT TCTAATGACA GAGAGTTTTT 6600
6601 GGATGGTCAC ATAGATCTTG GCCTTTTACT TTTGATGGAG GGTTGCAGAC GATCATTGAT 6660
6661 GAATGGAGGG CTTTGTTTTT AAGAATCTTA TTAGTGGGTA GTGGTTACTA GTTAGATGTC 6720
6721 TATCAATCTA TAGATAAAAA TAATGTTGTA TATCAAAGGA TTTTTGTTCT TCGCTTTCTT 6780
6781 ATGCCCAGTC ATTTTCTGAA TTCACGCGTT TTAATTAACC AATTCGTAAT CATGGTCATA 6840
6841 GCTGTTTCCT GTGTGAAATT GTTATCCGCT CACAATTCCA CACAACATAC GAGCCGGAAG 6900
6901 CATAAAGTGT AAAGCCTGGG GTGCCTAATG AGTGAGCTAA CTCACATTAA TTGCGTTGCG 6960
6961 CTCACTGCCC GCTTTCCAGT CGGGAAACCT GTCGTGCCAG CTGCATTAAT GAATCGGCCA 7020
7021 ACGCGCGGGG AGAGGCGGTT TGCGTATTGG AGCTTGAGCT TTAGACAACT TAATAACACA 7080
7081 TTGCGGACGT TTTTAATGTA CTGAATTAAC GCCGAATTGC TCTAGCATTC GCCATTCAGG 7140
7141 CTGCGCAACT GTTGGGAAGG GCGATCGGTG CGGGCCTCTT CGCTATTACG CCAGCTGGCG 7200
7201 AAAGGGGGAT GTGCTGCAAG GCGATTAAGT TGGGTAACGC CAGGGTTTTC CCAGTCACGA 7260
7261 CGTTGTAAAA CGACGGCCAG TGCCAAGCTG GCGATCGCTT TGGCGCGCCA AGCTTTTTGT 7320
7321 CTAGTTGGCA GCCTAATTAA TTCTATGGAA ACCAGGTGAC ATGGAGGGTT GGGGACATGG 7380
7381 TGGAAAAAAC CGGAACGGGC CGACAGTTCA ACCGGAAAAA ACCAGAACCC GTTCAGTTCA 7440
7441 AAAGAAAGAC CGGACATGCA TATGACCCGC TTTGAACCGG CAGAACCGGT CGGTTTTTCT 7500
7501 ATGAACCGGT CATTAAACCG TCCCCGGTTA GACCGAACAA GCCACAATAA TCTTGAAATG 7560
7561 GGCCTTGATG TGGCCCAATT GGTCTGCCTA GAGCGTTTTG GTTGGCAAAA ATCAATCTCC 7620
7621 TATTCTCGGC ACGTGTGATA TACAATGGTA AGTGAGATAT ACAATTCTCG GCACGGCTAC 7680
7681 ATTACAAGGT GTCGCATTGT GTCAATGTTT GGTTAATTTG CTAGATTCAC ATAATACATG 7740
7741 CCAGGAAGTT CAGAACAATG TGTTGCCTTT CACCGGAAAA CTTTGTTGGA GCAAATGCCT 7800
7801 TCTTCTTTTT TGCTTCTGCT TCTTGAGTCC ATGTGGAGGA AGCAGTAGAT AGCTGATGAT 7860
7861 ATCAGGATTC CTTCTGTGTC TGTGTAGGTG TAGCAACACC ACTATAATTT TTATTTAGCA 7920
7921 ACACAATATC AATTTGGTCT ATAAAAGTAT GAATTAAATC AATCCCCAAC CACAATTAGA 7980
7981 GTAAGTTGGT GAGTTATTGT AAAGCTCTGC AAAGTTAATT TAAAAGTTAT TGCATTAACT 8040
8041 TATTTCGTAT CACAAACAAG TTTTCACAAG AGTATTAATG GAACAATGAA AACCATTGAA 8100
8101 CATACTATAA TTTTTTTTCT TACTGAAATT ATATAATTCA AAGAGCATAA ACCCACACAG 8160
8161 TCGTAAAGTT CCACGTGTAG TGCATTATCA AAATAATAGC TTACAAAACA TAACAAACTT 8220
8221 AGTTTCAAAA GTTGCAATCC TTATCACATT GACACATAAA GTGAGCGATG AGTCATGTCA 8280
8281 TTATTTTTTT GCTCACCATC ATGTATATAT GATGGGCATA AAAGTTACTT TGATGATGAT 8340
8341 ATCAAAGAAC ATTTTTAGGT GCACCTAACA GAATATCCAA ATAATATGAC TCACTTAGAT 8400
8401 CCTAATATAG CATCAAGCAA AACTAACACT CTAAAGCAAC CGATAGGGAA ACATCTATAA 8460
8461 ATAGACAAGC ATAATGAAAA CCCTCCTCAT CCTTCACACA ATTCAAACAT TATAGTTGAA 8520
8521 GCATAGTAGT AGAATCCTAC AAAATCTAGT ATTGTAGAAT CAGCAATGGC AGCATACACC 8580

8581 AGCAAGATCT TTGCCCTGTT TGCCTTAATT GCTCTTTCTG CAAGTGCCAC TACTGCATCT 8640
8641 AGAACACCTC AACAGATTAC TGATTTGTGT GCAGAATACC ACAACACACA GATCCACACC 8700
8701 CTCAATGATA AGATTTTCTC TTATACAGAA TCTCTAGCTG GAAAGAGAGA GATGGCTATC 8760
8761 ATTACTTTCA AGAATGGTGC AACTTTCCAA GTAGAAGTAC CAGGCAGTCA ACATATAGAT 8820
8821 TCACAAAAGA AGGCAATTGA AAGGATGAAG GATACCCTGA GGATTGCATA TCTTACTGAA 8880
8881 GCTAAAGTTG AAAAGCTATG TGTATGGAAT AACAAGACTC CTCATGCAAT TGCCGCAATT 8940
8941 AGTATGGCAA ATTGAGAGCT CATTGTAATA GTATAATGGT TCAAATGTTA AAAATAAAGT 9000
9001 CATGCATCAT CATGCGTGAC AGTTGAAACT TGATGTCATA TAAATCTAAA TAAAATCACC 9060
9061 TATTTAAATA GCATTCATGT ATGAGGTTGC ATTATCATAG CTAATTACCA TCACAAAGAA 9120
9121 CTTTACAATT ACTATGTGCA TGCATTTGAT CCTAAGCTAC TTTGGCTATT AGATACAAAT 9180
9181 GGAGTGTATT AAGCAAGTCC AACTTTTCAT TCTAATAGGA ACAAACTTGA AGCACATATG 9240
9241 TCAATTACTA TATCCCCAAC AATTGATACA CTATAGCAGT GGTTGCAATG GCAGCTGCAG 9300
9301 GAATTGCTAG TGAACCCAAC AGCCCATATA ACTGAATTGA AGCCATGTCA GACATCCGTA 9360
9361 ATGAACAACA AATAATTCCA AATAGTGCAT AACCAATAAA TACTTAAAGA TTTGTCTTTC 9420
9421 AACACTAATG TTTGCATTTC ATGCAATTGT ATTGCTCTGG AAGCACCTTT TAACAGGTTG 9480
9481 TATCATCACA TGCATTACAT TGGAGGATAA GGATATAAGA GATCATATAT AGAATAGAGG 9540
9541 ATATGGTGTT TTAGAAAAAG AAGATGAAGA ATATACATTG TGAAACAGTG CATGATATAG 9600
9601 GCACATCACT TAATACATTA AAAGCACTGC CAGAAATCTA TGTTGGTGAA CAAATGCTCA 9660
9661 CTGGTTGTCT CACTTATATC ACCATTCACC AAGTTCACCG TATGTAACAA GCACTTGGCC 9720
9721 CCATGAATTC ACGCGCCAAG CTTGTAGTGA ACGGTCTGGA CAGAGATTCG TCGCGTCGTC 9780
9781 AGCGGAAGGA GGTGCTTGAA TCCCTGATGC TGCCGGTTCT CTGTGTGTTT TTGGCTTGTG 9840
9841 AGATGGGGTT TAGTCAGTGC AATTTCAGTG GATTTTGCTC AACTCGGTTG GATTGGCACG 9900
9901 AGATTGGTTC CGCGTGTGAC CGTTTGTTGG AGAACATGAA ATCGCGTTGC TCGTTCTGGT 9960
9961 TTGGATGCTC TGTTTTCTGA ACTAGGTTGC TCGAATTGAA TCTGATTGGT TTATGTCTAC 10020
10021 CTCCGAGTAT CAAGTAAGTT CAGATGAGCG GTTAGCTCGT TTTCAGAGCT AGACGGGATC 10080
10081 ACGATCTGGG AATACAATTT TTATGAGGCA AATGCAGCAA ATATAAAGTG ATCTAACTAA 10140
10141 TGCCACTTGA TATTATGATG AACATGTTGT TGTGAAACTG GAAAATGCAT GCCAAATTGC 10200
10201 CAACCATATA CATCTCATTA GTAGAGTAAC TTCGTGACAG TGAAAGAAAA CTTCATTCTA 10260
10261 GCTGAGGGGA GATCACATTG AAGGTATTCG TGATATAATA TGTTAGCAAG ATAAGTAGCT 10320
10321 AGGATGGTTT ATTCATAGCA CATTGGTACC TGGCAGCATG TCAGATAGAT ATTTTCTATT 10380
10381 TCAGCAATAT ATTGATCTTA CCTTGTGCAA CAGTTTTCTC CATCCAAGTT CAGTGAAAAT 10440
10441 CATTTGTCCT TGAACTTATC TACTCAAGCG ATGAACAATA CTCATACACA TGAGCATATG 10500
10501 AATCAGTTCT ATCTTCTATG AAGCCTTAAC CCTAGATTTA TCACAATCTA GTCATCTAGT 10560
10561 CCGGCAGACC ACATTGAACT CCATTGCAAT TTGTGAGTTG TTGTATGGCT TCGGAATGGG 10620
10621 GTCGAATCTT TCTTCAATCC AGGATGGAAA TTTTGAGATC TATACATCAC GTTTGGTACA 10680
10681 TCTCTACTAC TCTAGTACTA AAAACATGAA GATTCTGAAC ATGGACATTG CATAGTTCTC 10740

10741 CATCCCAATT CACCCTGCAG TGATCGCTGC ACTGGATAAT TATAATATCA GTTAAAATTG 10800
10801 AAAATAATGC AACTTCATAC TTGCATGGTG TCAGTAGTGC CTGCCTAAGA AATGTGTCTT 10860
10861 GTCATAATAT GATTACATGA AATATGTTTA CTTCCTCGTT TCTCTTTATT TGTAAGATAA 10920
10921 AGAACTAGAT ATGTGGAAAG TAGGATAGCA AAGAGTATGG CCAAACTCTA ATCTTTGCTT 10980
10981 TATTTTTTGG GATGGACCCA AAATTTGTTT CTCCTTTACT TCTTTCCCTT TACAACAATG 11040
11041 TTCTTTACTT CCAATTCTTA TTAACAAAAC TCCAAATACA TGCCAAACTG CATATGTATG 11100
11101 TATGCTATTA AGGCACATTT ACAAAGCTCC AAGTTTACCT ACTCAATCAT TCACATATGG 11160
11161 CGATGACTCA AACTCTTAAT TGTTATCTGT GTAAGCTGTG ACTTGTGTAA CACATTCTAC 11220
11221 AAGTCCCATA CCAATTCTGT TCACAAAAGT TTCTTTGTCC AGCTCATAAT TTACAAAACT 11280
11281 GCAAAATGCC AAAGCAATCT GGCACAACCT TATCATCATA TTTTCTTTCC ACGCATTAAA 11340
11341 GCACTGGCAG AATTATCTTT GTGTAGATAT TCCAAAAGTA TTGGTTGAAT AAATGTCCAA 11400
11401 ATAAATTCCA TGCCTCATGA TTTCCAGCTT ATGTGGCCTC CACTAGGTGG TTTTGCAAAG 11460
11461 GCCAAACTCT TTCCTGGCTT ACACAGCTAC CAGCATGTAT AAATAGGCCC CTAGGCAACC 11520
11521 ATTATTCCAT CATCCTCAAC AATATTGTCT ACACCATCTG GAATCTTGTT TAACACTAGA 11580
11581 ATTGTAGAAT CAGCATCTAG AAGGGCAACC GGGGAAGGTT AAACCCATAG AACCTCTCCC 11640
11641 TTGGATGATG TAGACCACGC CAGGAATATT TGTGTATCGA GGTACCAAAA GGCCTTGAGG 11700
11701 CTGAATGACA CGTCGGATCA CAAAAGTACC CGTGCACTGG AATAATTCAT TCTTCTCATC 11760
11761 GAAGTACTCA GTCACCCCAG CTTCTGACCT CACTTTCCGA AGTGGTTCAA ATGCTTGTAG 11820
11821 TCTATCAAAA CTAGAGCATG CAACAATAGC AAGGAGAGCA AATACGAAAA TGATCTTCAT 11880
11881 ACTAGACCCA GATCTCAGAA GAATGGTCCG TGCCTTAAAA CTTTCCCCAA CCGTGCTAGT 11940
11941 TTATGTTGTG ACTGTCTGCC TCTCTCAGTT TACTTGGATG CATTGACAAC ATCCTTTTTT 12000
12001 GCTATTACTC GTATTTGCTC TATAGCTGGT GGCATATCTC ATGTTGAAAT TTGCCCTTTT 12060
12061 AATCCAAAAT TGGATGTAAT TGAAAGAATC CTACGTGGTA GTTATTTGGA TTTTGGTGTG 12120
12121 AAAAAAAATA GCCTTGTTAG AAGAAGCAAA ATTGGATTTA GTTAAAAGGA TACTAGATGG 12180
12181 TGTTATTTGG ATTTTGGTGC AAATCAAATT AGGAGGTTGG TTTTATTCAA GTTAAAGTTT 12240
12241 GTTTTAAAAA AATTCTCCTA AAAAGATAGA TACTAGATTT GCATATATGC ATTGAAAATT 12300
12301 ACATCTTCGC TTGGCGGTTA TACTTTTAGT CCCTCTAAAT TGTTCAATCA TTTATGATGA 12360
12361 AAAGGAAAAT CATTTTATAT CACAAAGTAT TTATGATGAA AGGGGAAAAA TATTCTGCAT 12420
12421 GGGTTTGAAC AAAATACGTG GATTGGTGTA GCCTTAACAT ACTTGAAAAG GGTATGATGT 12480
12481 TGATGTAGTG CCCACATGGT GTCGCTTGAC ATTAAAACGA TATGCAGTCA GGATTGAGGA 12540
12541 ACATTGCTGA CAATTTACTA TCGCTGTCTG TGTTGACCAC AATAATTCAG ATGTACCATC 12600
12601 CTATCTTCTA ACTAGAAAGA TGCATGGAAG TTTCTTACAT TATTTCCAGC ACTTGAAATT 12660
12661 TTAGTGAAAT ATCATTAAAA CATAACCACT TACTTTGCTG TGATATGAAA TAAATGTTTT 12720
12721 ATTTCTTGGA AAGTGGTATA TTCATATATT CTTACAGTAA ATTTATTGAT TTTCTTTTCA 12780
12781 TTTATTTCTA AATTTTAACC ACCCTTTTGG TAGCTTAAGG AAAATTGTAT GTTTGACAGT 12840
12841 CCTGTTTTCT GTTGTTTCAT CCCTCCAGGA AAACCAGCTA CTAGTATGAA GATCATTTTC 12900

12901 GTATTTGCTC TCCTTGCTAT TGTTGCATGC TCTAGTTTTG ATAGACTACA AGCATTTGAA 12960

12961 CCACTTCGGA AAGTGAGGTC AGAAGCTGGG GTGACTGAGT ACTTCGATGA GAAGAATGAA 13020

13021 TTATTCCAGT GCACGGGTAC TTTTGTGATC CGACGTGTCA TTCAGCCTCA AGGCCTTTTG 13080

13081 GTACCTCGAT ACACAAATAT TCCTGGCGTG GTCTACATCA TCCAAGGGAG AGGTTCTATG 13140

13141 GGTTTAACCT TCCCCGGTTG CCCTTCTAGT GGATCCCCGG GTACCGAGCT CAAACGTTGG 13200

13201 TTACATGTAC TCTAGTAATA AGGTGTTGCA TACTATCGTG TGCAAACACT AGAAATAAGA 13260

13261 ACCATTGAAT AAAATATCAA TCATTTTCAG ACTTGCAAAT ATTGGGTATT TGGATTTCTG 13320

13321 TCCCATGTCC CTCTTGAAAG CCATGCTGTA CATGTTGGAG TTCCCCCTTG GACCCAACCT 13380

13381 ACTCCATGCT CCCATGTTGA TCTTAAATTC CCTGTTCCCC CAGAGCATGT AAATTTTCTT 13440

13441 ATGCTAATCA GAGCAAGCTC GATGTCTCAT TAACATATCC CTATTTGATC AATGTTGTGG 13500

13501 TTGTTTCAAA ATGTATTGGT GATAAGTGGT AACAGTGTAC TTCTGGCATA ATGGATAATA 13560

13561 AGTGGCGGAG GTTTGTGAAG ATAAGAGTTG GAAACAGAGC TGCTGTGTAT GTTCTAATGA 13620

13621 CAGAGAGTTT TTGGATGGTC ACATAGATCT TGGCCTTTTA CTTTTGATGG AGGGTTGCAG 13680

13681 ACGATCATTG ATGAATGGAG GGCTTTGTTT TTAAGAATCT TATTAGTGGG TAGTGGTTAC 13740

13741 TAGTTAGATG TCTATCAATC TATAGATAAA AATAATGTTG TATATCAAAG GATTTTTGTT 13800

13801 CTTCGCTTTC TTATGCCCAG TCATTTTCTG AATTCACGCG TTTTAATTAA CCAATTCGTA 13860

13861 ATCATGGTCA TAGCTGTTTC CTGTGTGAAA TTGTTATCCG CTCACAATTC CACACAACAT 13920

13921 ACGAGCCGGA AGCATAAAGT GTAAAGCCTG GGGTGCCTAA TGAGTGAGCT AACTCACATT 13980

13981 AATTGCGTTG CGCTCACTGC CCGCTTTCCA GTCGGGAAAC CTGTCGTGCC AGCTGCATTA 14040

14041 ATGAATCGGC CAACGCGCGG GGAGAGGCGG TTTGCGTATT GGAGCTTGAG CTTGGATCAG 14100

14101 ATTGTCGTTT CCCGCCTTCA GTTTAAACTA TCAGTGTTTG A

[The sequence of transgene on Chromosome 12]

1 GCAACTGTTG GGAAGGGCGA TCGGTGCGGG CCTCTTCGCT ATTACGCCAG CTGGCGAAAG 60
61 GGGGATGTGC TGCAAGGCGA TTAAGTTGGG TAACGCCAGG GTTTTCCCAG TCACGACGTT 120
121 GTAAAACGAC GGCCAGTGCC AAGCTGGCGA TCGCTTTGGC GCGCCAAGCT TTTTGTCTAG 180
181 TTGGCAGCCT AATTAATTCT ATGGAAACCA GGTGACATGG AGGGTTGGGG ACATGGTGGA 240
241 AAAAACCGGA ACGGGCCGAC AGTTCAACCG GAAAAAACCA GAACCCGTTC AGTTCAAAAG 300
301 AAAGACCGGA CATGCATATG ACCCGCTTTG AACCGGCAGA ACCGGTCGGT TTTTCTATGA 360
361 ACCGGTCATT AAACCGTCCC CGGTTAGACC GAACAAGCCA CAATAATCTT GAAATGGGCC 420
421 TTGATGTGGC CCAATTGGTC TGCCTAGAGC GTTTTGGTTG GCAAAAATCA ATCTCCTATT 480
481 CTCGGCACGT GTGATATACA ATGGTAAGTG AGATATACAA TTCTCGGCAC GGCTACATTA 540
541 CAAGGTGTCG CATTGTGTCA ATGTTTGGTT AATTTGCTAG ATTCACATAA TACATGCCAG 600
601 GAAGTTCAGA ACAATGTGTT GCCTTTCACC GGAAAACTTT GTTGGAGCAA ATGCCTTCTT 660
661 CTTTTTTGCT TCTGCTTCTT GAGTCCATGT GGAGGAAGCA GTAGATAGCT GATGATATCA 720
721 GGATTCCTTC TGTGTCTGTG TAGGTGTAGC AACACCACTA TAATTTTTAT TTAGCAACAC 780
781 AATATCAATT TGGTCTATAA AAGTATGAAT TAAATCAATC CCCAACCACA ATTAGAGTAA 840
841 GTTGGTGAGT TATTGTAAAG CTCTGCAAAG TTAATTTAAA AGTTATTGCA TTAACTTATT 900
901 TCGTATCACA AACAAGTTTT CACAAGAGTA TTAATGGAAC AATGAAAACC ATTGAACATA 960
961 CTATAATTTT TTTTCTTACT GAAATTATAT AATTCAAAGA GCATAAACCC ACACAGTCGT 1020
1021 AAAGTTCCAC GTGTAGTGCA TTATCAAAAT AATAGCTTAC AAAACATAAC AAACTTAGTT 1080
1081 TCAAAAGTTG CAATCCTTAT CACATTGACA CATAAAGTGA GCGATGAGTC ATGTCATTAT 1140
1141 TTTTTTGCTC ACCATCATGT ATATATGATG GGCATAAAAG TTACTTTGAT GATGATATCA 1200
1201 AAGAACATTT TTAGGTGCAC CTAACAGAAT ATCCAAATAA TATGACTCAC TTAGATCCTA 1260
1261 ATATAGCATC AAGCAAAACT AACACTCTAA AGCAACCGAT AGGGAAACAT CTATAAATAG 1320
1321 ACAAGCATAA TGAAAACCCT CCTCATCCTT CACACAATTC AAACATTATA GTTGAAGCAT 1380
1381 AGTAGTAGAA TCCTACAAAA TCTAGTATTG TAGAATCAGC AATGGCAGCA TACACCAGCA 1440
1441 AGATCTTTGC CCTGTTTGCC TTAATTGCTC TTTCTGCAAG TGCCACTACT GCATCTAGAA 1500
1501 CACCTCAACA GATTACTGAT TTGTGTGCAG AATACCACAA CACACAGATC CACACCCTCA 1560
1561 ATGATAAGAT TTTCTCTTAT ACAGAATCTC TAGCTGGAAA GAGAGAGATG GCTATCATTA 1620
1621 CTTTCAAGAA TGGTGCAACT TTCCAAGTAG AAGTACCAGG CAGTCAACAT ATAGATTCAC 1680
1681 AAAAGAAGGC AATTGAAAGG ATGAAGGATA CCCTGAGGAT TGCATATCTT ACTGAAGCTA 1740
1741 AAGTTGAAAA GCTATGTGTA TGGAATAACA AGACTCCTCA TGCAATTGCC GCAATTAGTA 1800
1801 TGGCAAATTG AGAGCTCATT GTAATAGTAT AATGGTTCAA ATGTTAAAAA TAAAGTCATG 1860
1861 CATCATCATG CGTGACAGTT GAAACTTGAT GTCATATAAA TCTAAATAAA ATCACCTATT 1920
1921 TAAATAGCAT TCATGTATGA GGTTGCATTA TCATAGCTAA TTACCATCAC AAAGAACTTT 1980
1981 ACAATTACTA TGTGCATGCA TTTGATCCTA AGCTACTTTG GCTATTAGAT ACAAATGGAG 2040
2041 TGTATTAAGC AAGTCCAACT TTTCATTCTA ATAGGAACAA ACTTGAAGCA CATATGTCAA 2100

2101 TTACTATATC CCCAACAATT GATACACTAT AGCAGTGGTT GCAATGGCAG CTGCAGGAAT 2160
2161 TGCTAGTGAA CCCAACAGCC CATATAACTG AATTGAAGCC ATGTCAGACA TCCGTAATGA 2220
2221 ACAACAAATA ATTCCAAATA GTGCATAACC AATAAATACT TAAAGATTTG TCTTTCAACA 2280
2281 CTAATGTTTG CATTTCATGC AATTGTATTG CTCTGGAAGC ACCTTTTAAC AGGTTGTATC 2340
2341 ATCACATGCA TTACATTGGA GGATAAGGAT ATAAGAGATC ATATATAGAA TAGAGGATAT 2400
2401 GGTGTTTTAG AAAAAGAAGA TGAAGAATAT ACATTGTGAA ACAGTGCATG ATATAGGCAC 2460
2461 ATCACTTAAT ACATTAAAAG CACTGCCAGA AATCTATGTT GGTGAACAAA TGCTCACTGG 2520
2521 TTGTCTCACT TATATCACCA TTCACCAAGT TCACCGTATG TAACAAGCAC TTGGCCCCAT 2580
2581 GAATTCACGC GCCAAGCTTG TAGTGAACGG TCTGGACAGA GATTCGTCGC GTCGTCAGCG 2640
2641 GAAGGAGGTG CTTGAATCCC TGATGCTGCC GGTTCTCTGT GTGTTTTTGG CTTGTGAGAT 2700
2701 GGGGTTTAGT CAGTGCAATT TCAGTGGATT TTGCTCAACT CGGTTGGATT GGCACGAGAT 2760
2761 TGGTTCCGCG TGTGACCGTT TGTTGGAGAA CATGAAATCG CGTTGCTCGT TCTGGTTTGG 2820
2821 ATGCTCTGTT TTCTGAACTA GGTTGCTCGA ATTGAATCTG ATTGGTTTAT GTCTACCTCC 2880
2881 GAGTATCAAG TAAGTTCAGA TGAGCGGTTA GCTCGTTTTC AGAGCTAGAC GGGATCACGA 2940
2941 TCTGGGAATA CAATTTTTAT GAGGCAAATG CAGCAAATAT AAAGTGATCT AACTAATGCC 3000
3001 ACTTGATATT ATGATGAACA TGTTGTTGTG AAACTGGAAA ATGCATGCCA AATTGCCAAC 3060
3061 CATATACATC TCATTAGTAG AGTAACTTCG TGACAGTGAA AGAAAACTTC ATTCTAGCTG 3120
3121 AGGGGAGATC ACATTGAAGG TATTCGTGAT ATAATATGTT AGCAAGATAA GTAGCTAGGA 3180
3181 TGGTTTATTC ATAGCACATT GGTACCTGGC AGCATGTCAG ATAGATATTT TCTATTTCAG 3240
3241 CAATATATTG ATCTTACCTT GTGCAACAGT TTTCTCCATC CAAGTTCAGT GAAAATCATT 3300
3301 TGTCCTTGAA CTTATCTACT CAAGCGATGA ACAATACTCA TACACATGAG CATATGAATC 3360
3361 AGTTCTATCT TCTATGAAGC CTTAACCCTA GATTTATCAC AATCTAGTCA TCTAGTCCGG 3420
3421 CAGACCACAT TGAACTCCAT TGCAATTTGT GAGTTGTTGT ATGGCTTCGG AATGGGGTCG 3480
3481 AATCTTTCTT CAATCCAGGA TGGAAATTTT GAGATCTATA CATCACGTTT GGTACATCTC 3540
3541 TACTACTCTA GTACTAAAAA CATGAAGATT CTGAACATGG ACATTGCATA GTTCTCCATC 3600
3601 CCAATTCACC CTGCAGTGAT CGCTGCACTG GATAATTATA ATATCAGTTA AAATTGAAAA 3660
3661 TAATGCAACT TCATACTTGC ATGGTGTCAG TAGTGCCTGC CTAAGAAATG TGTCTTGTCA 3720
3721 TAATATGATT ACATGAAATA TGTTTACTTC CTCGTTTCTC TTTATTTGTA AGATAAAGAA 3780
3781 CTAGATATGT GGAAAGTAGG ATAGCAAAGA GTATGGCCAA ACTCTAATCT TTGCTTTATT 3840
3841 TTTTGGGATG GACCCAAAAT TTGTTTCTCC TTTACTTCTT TCCCTTTACA ACAATGTTCT 3900
3901 TTACTTCCAA TTCTTATTAA CAAAACTCCA AATACATGCC AAACTGCATA TGTATGTATG 3960
3961 CTATTAAGGC ACATTTACAA AGCTCCAAGT TTACCTACTC AATCATTCAC ATATGGCGAT 4020
4021 GACTCAAACT CTTAATTGTT ATCTGTGTAA GCTGTGACTT GTGTAACACA TTCTACAAGT 4080
4081 CCCATACCAA TTCTGTTCAC AAAAGTTTCT TTGTCCAGCT CATAATTTAC AAAACTGCAA 4140
4141 AATGCCAAAG CAATCTGGCA CAACCTTATC ATCATATTTT CTTTCCACGC ATTAAAGCAC 4200
4201 TGGCAGAATT ATCTTTGTGT AGATATTCCA AAAGTATTGG TTGAATAAAT GTCCAAATAA 4260

4261 ATTCCATGCC TCATGATTTC CAGCTTATGT GGCCTCCACT AGGTGGTTTT GCAAAGGCCA 4320
4321 AACTCTTTCC TGGCTTACAC AGCTACCAGC ATGTATAAAT AGGCCCCTAG GCAACCATTA 4380
4381 TTCCATCATC CTCAACAATA TTGTCTACAC CATCTGGAAT CTTGTTTAAC ACTAGAATTG 4440
4441 TAGAATCAGC ATCTAGAAGG GCAACCGGGG AAGGTTAAAC CCATAGAACC TCTCCCTTGG 4500
4501 ATGATGTAGA CCACGCCAGG AATATTTGTG TATCGAGGTA CCAAAAGGCC TTGAGGCTGA 4560
4561 ATGACACGTC GGATCACAAA AGTACCCGTG CACTGGAATA ATTCATTCTT CTCATCGAAG 4620
4621 TACTCAGTCA CCCCAGCTTC TGACCTCACT TTCCGAAGTG GTTCAAATGC TTGTAGTCTA 4680
4681 TCAAAACTAG AGCATGCAAC AATAGCAAGG AGAGCAAATA CGAAAATGAT CTTCATACTA 4740
4741 GTAGCTGGTT TTCCTGGAGG GATGAAACAA CAGAAAACAG GACTGTCAAA CATACAATTT 4800
4801 TCCTTAAGCT ACCAAAAGGG TGGTTAAAAT TTAGAAATAA ATGAAAAGAA AATCAATAAA 4860
4861 TTTACTGTAA GAATATATGA ATATACCACT TTCCAAGAAA TAAAACATTT ATTTCATATC 4920
4921 ACAGCAAAGT AAGTGGTTAT GTTTTAATGA TATTTCACTA AAATTTCAAG TGCTGGAAAT 4980
4981 AATGTAAGAA ACTTCCATGC ATCTTTCTAG TTAGAAGATA GGATGGTACA TCTGAATTAT 5040
5041 TGTGGTCAAC ACAGACAGCG ATAGTAAATT GTCAGCAATG TTCCTCAATC CTGACTGCAT 5100
5101 ATCGTTTTAA TGTCAAGCGA CACCATGTGG GCACTACATC AACATCATAC CCTTTTCAAG 5160
5161 TATGTTAAGG CTACACCAAT CCACGTATTT TGTTCAAACC CATGCAGAAT ATTTTTCCCC 5220
5221 TTTCATCATA AATACTTTGT GATATAAAAT GATTTTCCTT TTCATCATAA ATGATTGAAC 5280
5281 AATTTAGAGG GACTAAAAGT ATAACCGCCA AGCGAAGATG TAATTTTCAA TGCATATATG 5340
5341 CAAATCTAGT ATCTATCTTT TTAGGAGAAT TTTTTTAAAA CAAACTTTAA CTTGAATAAA 5400
5401 ACCAACCTCC TAATTTGATT TGCACCAAAA TCCAAATAAC ACCATCTAGT ATCCTTTTAA 5460
5461 CTAAATCCAA TTTTGCTTCT TCTAACAAGG CTATTTTTTT TCACACCAAA ATCCAAATAA 5520
5521 CTACCACGTA GGATTCTTTC AATTACATCC AATTTTGGAT TAAAAGGGCA AATTTCAACA 5580
5581 TGAGATATGC CACCAGCTAT AGAGCAAATA CGAGTAATAG CAAAAAAGGA TGTTGTCAAT 5640
5641 GCATCCAAGT AAACTGAGAG AGGCAGACAG TCA 5700

Figure S1 Sequences of transgene regions of NBS on chromosomes 3 and 12. Because the sequences of transgenes of MSB and NSB were completely the same, it was shown only sequence of NBS. Part of the right border is double underlined. Part of the left border is underlined. CTB coding region is shown in red. RNAi regions, including the trigger sequences of 13-kDa prolamin and glutelin B, are shaded in gray. Residues shaded in yellow show corrected residues after amendment to our previous MSB sequence data.
