# Supplementary material for: Comparative whole-genome and proteomics analyses of the next seed bank and the original master seed bank of MucoRice-CTB 51A line, a rice-based oral cholera vaccine
Source: BMC Genomics. 2021 Jan 19;22:59. doi: 10.1186/s12864-020-07355-7 (PMC7814724; doi:10.1186/s12864-020-07355-7)
Supplement: Supplementary file 3 — Additional file 3: Table S3. List of salt-soluble proteins of MucoRice-CTB identified in both MSB and NSB by shot-gun MS/MS analysis [file 12864_2020_7355_MOESM3_ESM.pdf]

Table S3. The list of salt-soluble proteins of MucoRice-CTB identified in both MSB and NSB by Shot-gun MS analysis

			MSB				NSB				
No.	Accession	Description	# PSMs	# AAs	MW [kDa]	calc. pI	# PSMs	# AAs	MW [kDa]	calc. pI	#PSMs NSB/MSB
1	Q75GX9.1	RecName: Full=63 kDa globulin-like protein; AltName: Allergen=Ory s GLP63; Flags: Precursor	2548	562	63.4	8.13	2358	562	63.4	8.13	0.925
2	XP_015628337.1	cupincin [Oryza sativa Japonica Group]	984	470	52.1	7.25	852	470	52.1	7.25	0.866
3	AAD10375.1	globulin-like protein, partial [Oryza sativa]	1171	461	51.7	10.81	860	461	51.7	10.81	0.734
4	BAF11185.1	Os03g0197300 [Oryza sativa Japonica Group]	315	601	68.2	5.71	444	601	68.2	5.71	1.410
5	AAA72362.1	unnamed protein product [Oryza sativa Japonica Group]	487	174	19.8	6.96	614	174	19.8	6.96	1.261
6	EAZ41388.1	hypothetical protein OsJ_25906 [Oryza sativa Japonica Group]	287	526	57.5	8.25	281	526	57.5	8.25	0.979
7	XP_015650705.1	lactoylglutathione lyase [Oryza sativa Japonica Group]	264	291	32.5	5.67	331	291	32.5	5.67	1.254
8	Q42971.2	RecName: Full=Enolase; AltName: Full=2-phospho-D-glycerate hydro-lyase; AltName: Full=2-phosphoglycerate dehydratase; AltName: Full=OSE1	171	446	47.9	5.57	152	446	47.9	5.57	0.889
9	XP_015637572.1	late embryogenesis abundant protein 19 [Oryza sativa Japonica Group]	156	200	20.5	6.25	116	200	20.5	6.25	0.744
10	XP_015613809.1	fructose-bisphosphate aldolase 3, cytoplasmic [Oryza sativa Japonica Group]	186	358	38.8	8.16	152	358	38.8	8.16	0.817
11	XP_015625618.1	heat shock 70 kDa protein BIP1 [Oryza sativa Japonica Group]	187	665	73.3	5.19	150	665	73.3	5.19	0.802
12	Q75KH3.2	RecName: Full=Glucose and ribitol dehydrogenase homolog	210	300	32.2	6.05	218	300	32.2	6.05	1.038
13	XP_015645300.1	1-Cys peroxiredoxin A [Oryza sativa Japonica Group]	182	220	24.0	6.43	148	220	24.0	6.43	0.813
14	CAA69949.1	lipid transfer protein [Oryza sativa]	148	116	11.4	9.13	59	116	11.4	9.13	0.399
15	XP_015625436.1	embryonic protein DC-8 [Oryza sativa Japonica Group]	128	471	47.3	6.87	156	471	47.3	6.87	1.219
16	XP_015645309.1	17kDa alpha-amylase/trypsin inhibitor 2 [Oryza sativa Japonica Group]	128	159	16.5	7.50	106	159	16.5	7.50	0.828
17	ACJ54890.1	heat shock protein 70 [Oryza sativa Japonica Group]	117	653	71.6	5.43	140	653	71.6	5.43	1.197
18	XP_015625199.1	16.9 kDa class I heat shock protein 1 [Oryza sativa Japonica Group]	87	150	16.9	6.61	96	150	16.9	6.61	1.103
19	XP_015650130.1	glyceraldehyde-3-phosphate dehydrogenase 1, cytosolic [Oryza sativa Japonica Group]	99	337	36.4	7.11	79	337	36.4	7.11	0.798
20	XP_008780653.1	16.9 kDa class I heat shock protein 2 [Phoenix dactylifera]	87	150	16.9	6.61	97	150	16.9	6.61	1.115
21	ACA50505.1	seed allergenic protein RAG2 [Oryza sativa Japonica Group]	121	166	17.8	8.03	135	166	17.8	8.03	1.116
22	EAZ04671.1	hypothetical protein OsI_26825 [Oryza sativa Indica Group]	68	234	23.7	6.20	75	234	23.7	6.20	1.103

23	XP_015611186.1	embryonic protein DC-8 [Oryza sativa Japonica Group]	56	241	24.5	6.40	55	241	24.5	6.40	0.982
24	XP_015615102.1	non-specific lipid-transfer protein 1-like [Oryza sativa Japonica Group]	120	117	11.6	9.80	36	117	11.6	9.80	0.300
25	XP_015632814.1	vicilin-like seed storage protein At2g28490 [Oryza sativa Japonica Group]	102	565	61.4	7.53	112	565	61.4	7.53	1.098
26	XP_015625382.1	glyceraldehyde-3-phosphate dehydrogenase 3, cytosolic [Oryza sativa Japonica Group]	93	337	36.5	7.88	73	337	36.5	7.88	0.785
27	BAA05537.1	WSI18 protein induced by water stress [Oryza sativa Japonica Group]	72	214	22.1	9.14	95	214	22.1	9.14	1.319
28	XP_015640019.1	ATP synthase subunit beta, mitochondrial [Oryza sativa Japonica Group]	84	552	58.9	6.37	54	552	58.9	6.37	0.643
29	XP_015640166.1	probable mediator of RNA polymerase II transcription subunit 37c [Oryza sativa Japonica Group]	99	646	70.8	5.21	119	646	70.8	5.21	1.202
30	XP_015645679.1	17kDa alpha-amylase/trypsin inhibitor 1 [Oryza sativa Japonica Group]	92	158	16.4	7.50	60	158	16.4	7.50	0.652
31	XP_015633414.1	16.9 kDa class I heat shock protein 3 [Oryza sativa Japonica Group]	71	149	16.9	7.42	79	149	16.9	7.42	1.113
32	XP_015613274.1	low-temperature-induced 65 kDa protein [Oryza sativa Japonica Group]	69	446	45.6	4.87	64	446	45.6	4.87	0.928
33	BAT15366.1	Os11g0701100, partial [Oryza sativa Japonica Group]	80	284	31.0	6.54	71	284	31.0	6.54	0.888
34	EEC74867.1	hypothetical protein OsI_10758 [Oryza sativa Indica Group]	77	445	48.0	5.47	80	445	48.0	5.47	1.039
35	ABD57308.1	UDP-glucose pyrophosphorylase [Oryza sativa Indica Group]	95	469	51.6	5.59	80	469	51.6	5.59	0.842
36	XP_015631876.1	pyruvate decarboxylase 2 [Oryza sativa Japonica Group]	66	605	64.7	5.81	44	605	64.7	5.81	0.667
37	ACV65034.1	alpha-amylase inhibitor [Oryza sativa Indica Group]	89	179	19.2	8.19	79	179	19.2	8.19	0.888
38	XP_015644193.1	phosphoglycerate kinase, cytosolic [Oryza sativa Japonica Group]	81	401	42.3	6.61	67	401	42.3	6.61	0.827
39	XP_015618966.1	probable mediator of RNA polymerase II transcription subunit 37c [Oryza sativa Japonica Group]	83	648	70.9	5.21	94	648	70.9	5.21	1.133
40	XP_015629108.1	late embryogenesis abundant protein 17 [Oryza sativa Japonica Group]	77	344	36.8	6.83	60	344	36.8	6.83	0.779
41	XP_015614294.1	malate dehydrogenase, cytoplasmic [Oryza sativa Japonica Group]	67	332	35.5	6.09	49	332	35.5	6.09	0.731
42	BAF12344.1	Os03g0432100 [Oryza sativa Japonica Group]	64	796	87.0	5.53	26	796	87.0	5.53	0.406
43	BAS73554.1	Os01g0663400, partial [Oryza sativa Japonica Group]	76	445	47.7	5.96	95	445	47.7	5.96	1.250
44	XP_015639340.1	aldose reductase [Oryza sativa Japonica Group]	66	318	35.6	6.80	69	318	35.6	6.80	1.045
45	AAX85991.1	protein disulfide isomerase [Oryza sativa Japonica Group]	85	512	56.8	5.06	70	512	56.8	5.06	0.824
46	XP_015649739.1	glycine-rich protein 2 [Oryza sativa Japonica Group]	42	197	18.7	6.76	30	197	18.7	6.76	0.714

47	XP_015630538.1	heat shock cognate 70 kDa protein 2 [Oryza sativa Japonica Group]	69	650	71.1	5.21	81	650	71.1	5.21	1.174
48	ADM86861.1	triosephosphate isomerase [Oryza sativa Japonica Group]	66	253	27.0	5.49	67	253	27.0	5.49	1.015
49	AAB23101.1	bifunctional subtilisin/alpha-amylase inhibitor, RASI [Oryza sativa=rice, seeds, bran, Peptide, 176 aa] [Oryza sativa]	71	176	18.7	9.04	56	176	18.7	9.04	0.789
50	XP_015635626.1	glyceraldehyde-3-phosphate dehydrogenase 2, cytosolic [Oryza sativa Japonica Group]	64	337	36.7	6.81	43	337	36.7	6.81	0.672
51	BAF14618.2	Os04g0404400 [Oryza sativa Japonica Group]	56	295	31.2	5.02	57	295	31.2	5.02	1.018
52	XP_015636388.1	ABA-inducible protein PHV A1 [Oryza sativa Japonica Group]	48	274	28.8	9.10	39	274	28.8	9.10	0.813
53	Q6YZX6.1	RecName: Full=Putative aconitate hydratase, cytoplasmic; Short=Aconitase; AltName: Full=Citrate hydro-lyase	54	898	98.0	6.01	57	898	98.0	6.01	1.056
54	EEE56924.1	hypothetical protein OsJ_06602 [Oryza sativa Japonica Group]	48	489	54.8	8.85	51	489	54.8	8.85	1.063
55	AAA57130.1	manganese superoxide dismutase [Oryza sativa]	52	231	24.9	7.02	57	231	24.9	7.02	1.096
56	XP_015625235.1	elongation factor 2 [Oryza sativa Japonica Group]	60	843	94.0	6.16	47	843	94.0	6.16	0.783
57	XP_015646664.1	seed allergenic protein RAG2-like [Oryza sativa Japonica Group]	68	160	17.3	8.34	101	160	17.3	8.34	1.485
58	EEE60487.1	hypothetical protein OsJ_13773 [Oryza sativa Japonica Group]	52	895	99.9	5.95	61	895	99.9	5.95	1.173
59	Q01881.2	RecName: Full=Seed allergenic protein RA5; AltName: Allergen=Ory s aA_TI; Flags: Precursor	62	160	17.3	8.03	58	160	17.3	8.03	0.935
60	Q8H8U5.1	RecName: Full=Protein IN2-1 homolog B; AltName: Full=Glutathione S-transferase GSTZ5	39	244	27.3	5.53	43	244	27.3	5.53	1.103
61	XP_015643985.1	late embryogenesis abundant protein D-34 [Oryza sativa Japonica Group]	40	277	27.9	4.36	42	277	27.9	4.36	1.050
62	XP_015629184.1	uncharacterized protein LOC4332421 [Oryza sativa Japonica Group]	47	139	15.0	5.86	56	139	15.0	5.86	1.191
63	BAA06876.1	aspartic protease [Oryza sativa]	55	509	54.1	5.26	48	509	54.1	5.26	0.873
64	AAB67852.1	osmotin, partial [Oryza sativa]	42	218	22.4	7.65	39	218	22.4	7.65	0.929
65	XP_015639252.1	fructose-bisphosphate aldolase 1, cytoplasmic [Oryza sativa Japonica Group]	55	358	38.8	7.33	64	358	38.8	7.33	1.164
66	ABC74439.1	differentiation embryo protein 31 [Oryza sativa Indica Group]	65	392	47.4	6.51	85	392	47.4	6.51	1.308
67	XP_015611801.1	protein disulfide isomerase-like 2-3 [Oryza sativa Japonica Group]	48	441	47.3	5.58	48	441	47.3	5.58	1.000
68	XP_015623194.1	poly [ADP-ribose] polymerase 3 isoform X8 [Oryza sativa Japonica Group]	44	831	92.3	5.63	55	831	92.3	5.63	1.250

69	XP_015630471.1	17.9 kDa class I heat shock protein [Oryza sativa Japonica Group]	33	161	17.9	6.06	23	161	17.9	6.06	0.697
70	XP_015621140.1	dehydrin Rab25 [Oryza sativa Japonica Group]	42	228	23.2	7.17	39	228	23.2	7.17	0.929
71	XP_015625227.1	peptidyl-prolyl cis-trans isomerase [Oryza sativa Japonica Group]	48	172	18.3	8.34	33	172	18.3	8.34	0.688
72	ABA92030.1	Alcohol dehydrogenase 1, putative, expressed [Oryza sativa Japonica Group]	49	345	37.5	6.65	35	345	37.5	6.65	0.714
73	XP_015622026.1	Bowman-Birk type bran trypsin inhibitor [Oryza sativa Japonica Group]	42	254	27.8	5.57	36	254	27.8	5.57	0.857
74	XP_015645223.1	alpha-amylase/trypsin inhibitor RA16 [Oryza sativa Japonica Group]	45	157	17.0	8.03	73	157	17.0	8.03	1.622
75	EEE69924.1	hypothetical protein OsJ_29787 [Oryza sativa Japonica Group]	42	399	45.1	4.97	48	399	45.1	4.97	1.143
76	NP_001105455.2	calmodulin [Zea mays]	25	149	16.8	4.27	24	149	16.8	4.27	0.960
77	XP_015640940.1	peroxiredoxin-2E-1, chloroplastic [Oryza sativa Japonica Group]	33	232	23.7	8.79	28	232	23.7	8.79	0.848
78	EEC71703.1	hypothetical protein OsI_04213 [Oryza sativa Indica Group]	39	559	60.7	5.60	34	559	60.7	5.60	0.872
79	XP_015612430.1	uncharacterized protein LOC4324175 [Oryza sativa Japonica Group]	34	83	8.5	4.97	44	83	8.5	4.97	1.294
80	AAD10378.1	Bowman-Birk type trypsin inhibitor [Oryza sativa]	32	136	15.4	6.13	23	136	15.4	6.13	0.719
81	P37833.1	RecName: Full=Aspartate aminotransferase, cytoplasmic; AltName: Full=Transaminase A	35	407	44.5	7.94	23	407	44.5	7.94	0.657
82	XP_015643982.1	pyrophosphate--fructose 6-phosphate 1-phosphotransferase subunit beta [Oryza sativa Japonica Group]	36	567	61.2	6.42	23	567	61.2	6.42	0.639
83	XP_015619851.1	5-methyltetrahydropteroyltriglutamate--homocysteine methyltransferase 2 [Oryza sativa Japonica Group]	43	766	84.6	6.30	50	766	84.6	6.30	1.163
84	XP_015619459.1	late embryogenesis abundant protein D-34 [Oryza sativa Japonica Group]	30	220	21.9	4.77	43	220	21.9	4.77	1.433
85	EAZ13499.1	hypothetical protein OsJ_03416 [Oryza sativa Japonica Group]	35	573	63.2	6.95	36	573	63.2	6.95	1.029
86	XP_015643120.1	probable alpha-glucosidase Os06g0675700 isoform X3 [Oryza sativa Japonica Group]	25	885	96.3	8.59	28	885	96.3	8.59	1.120
87	XP_015613091.1	lysosomal Pro-X carboxypeptidase [Oryza sativa Japonica Group]	29	517	57.4	5.31	10	517	57.4	5.31	0.345
88	CAA39535.1	chitinase, partial [Oryza sativa Japonica Group]	29	303	31.8	6.80	26	303	31.8	6.80	0.897
89	XP_015650698.1	alpha-amylase isozyme 3E [Oryza sativa Japonica Group]	31	437	48.7	5.63	17	437	48.7	5.63	0.548
90	XP_015632174.1	actin-depolymerizing factor 4 [Oryza sativa Japonica Group]	21	139	15.9	6.04	16	139	15.9	6.04	0.762

91	XP_015647572.1	thioredoxin H1 [Oryza sativa Japonica Group]	37	122	13.1	5.30	31	122	13.1	5.30	0.838
92	EAZ03220.1	hypothetical protein OsI_25368 [Oryza sativa Indica Group]	35	141	15.3	6.95	27	141	15.3	6.95	0.771
93	XP_015639465.1	malate dehydrogenase, mitochondrial [Oryza sativa Japonica Group]	34	340	35.4	8.10	46	340	35.4	8.10	1.353
94	XP_015635734.1	late embryogenesis abundant protein 18 [Oryza sativa Japonica Group]	21	122	12.6	9.52	26	122	12.6	9.52	1.238
95	XP_015620018.1	glycine-rich RNA-binding protein 2 [Oryza sativa Japonica Group]	21	162	16.1	6.74	20	162	16.1	6.74	0.952
96	XP_015631886.1	universal stress protein A-like protein isoform X4 [Oryza sativa Japonica Group]	15	182	18.9	6.93	11	182	18.9	6.93	0.733
97	XP_015621604.1	malate dehydrogenase, mitochondrial [Oryza sativa Japonica Group]	31	340	35.4	8.56	43	340	35.4	8.56	1.387
98	EEC78570.1	hypothetical protein OsI_18555 [Oryza sativa Indica Group]	29	502	52.7	7.75	19	502	52.7	7.75	0.655
99	CAA77235.1	reversibly glycosylated polypeptide [Oryza sativa Indica Group]	34	364	41.3	6.19	33	364	41.3	6.19	0.971
100	ABA93500.1	Adenosylhomocysteinase, putative, expressed [Oryza sativa Japonica Group]	32	450	49.3	6.27	21	450	49.3	6.27	0.656
101	Q65XA0.1	RecName: Full=Probable glutathione S-transferase DHAR1, cytosolic; AltName: Full=GSH-dependent dehydroascorbate reductase 1; Short=OsDHAR1; AltName: Full=Glutathione-dependent dehydroascorbate reductase 1	29	213	23.6	6.21	27	213	23.6	6.21	0.931
102	EEC75043.1	hypothetical protein OsI_11143 [Oryza sativa Indica Group]	31	256	28.5	6.04	34	256	28.5	6.04	1.097
103	AAN05528.1	mitochondrial chaperonin-60 [Oryza sativa Japonica Group]	26	574	60.8	5.87	23	574	60.8	5.87	0.885
104	EAY88627.1	hypothetical protein OsI_10103 [Oryza sativa Indica Group]	26	276	27.1	5.05	43	276	27.1	5.05	1.654
105	EEE62108.1	hypothetical protein OsJ_16892 [Oryza sativa Japonica Group]	29	359	38.2	6.16	20	359	38.2	6.16	0.690
106	XP_015617058.1	14-3-3-like protein GF14-D [Oryza sativa Japonica Group]	31	265	29.2	4.91	21	265	29.2	4.91	0.677
107	XP_015650889.1	betaine aldehyde dehydrogenase 2 [Oryza sativa Japonica Group]	19	503	54.6	5.45	19	503	54.6	5.45	1.000
108	XP_015614438.1	alanine aminotransferase 2 [Oryza sativa Japonica Group]	25	483	52.6	6.65	22	483	52.6	6.65	0.880
109	EAZ06557.1	hypothetical protein OsI_28805 [Oryza sativa Indica Group]	27	150	15.2	8.51	30	150	15.2	8.51	1.111
110	XP_015647859.1	elongation factor 1-delta 1 [Oryza sativa Japonica Group]	19	229	24.8	4.53	11	229	24.8	4.53	0.579

111	EEC83981.1	hypothetical protein OsI_30128 [Oryza sativa Indica Group]	24	361	38.5	6.39	25	361	38.5	6.39	1.042
112	EAZ00194.1	hypothetical protein OsI_22198 [Oryza sativa Indica Group]	32	317	35.7	6.74	24	317	35.7	6.74	0.750
113	XP_015614111.1	heme-binding protein 2 [Oryza sativa Japonica Group]	20	218	23.6	4.88	23	218	23.6	4.88	1.150
114	AAC78393.1	low molecular mass heat shock protein Oshsp18.0 [Oryza sativa Japonica Group]	18	160	18.0	7.42	19	160	18.0	7.42	1.056
115	XP_008780868.1	class II metallothionein-like protein 1A [Phoenix dactylifera]	25	87	8.5	7.33	38	87	8.5	7.33	1.520
116	XP_025878742.1	leucine aminopeptidase 2, chloroplastic [Oryza sativa Japonica Group]	21	532	55.1	5.80	13	532	55.1	5.80	0.619
117	A6N0M9.1	RecName: Full=Nucleoside diphosphate kinase 1; AltName: Full=Nucleoside diphosphate kinase I; Short=NDK I; Short=NDP kinase I; Short=NDPK I	30	149	16.8	6.80	27	149	16.8	6.80	0.900
118	XP_015620921.1	guanine nucleotide-binding protein subunit beta-like protein A [Oryza sativa Japonica Group]	19	334	36.2	6.44	20	334	36.2	6.44	1.053
119	BAS94548.1	Os05g0477900, partial [Oryza sativa Japonica Group]	15	83	8.1	9.10	12	83	8.1	9.10	0.800
120	BAT11451.1	Os10g0493600, partial [Oryza sativa Japonica Group]	19	369	40.8	7.12	9	369	40.8	7.12	0.474
121	XP_015617611.1	dehydrin Rab16D [Oryza sativa Japonica Group]	18	151	15.5	9.14	12	151	15.5	9.14	0.667
122	XP_015650060.1	60S acidic ribosomal protein P1 [Oryza sativa Japonica Group]	14	110	11.1	4.51	9	110	11.1	4.51	0.643
123	XP_015614147.1	nucleoside diphosphate kinase 1 [Oryza sativa Japonica Group]	19	151	16.8	7.50	15	151	16.8	7.50	0.789
124	XP_015622168.1	nascent polypeptide-associated complex subunit alpha-like protein 1 [Oryza sativa Japonica Group]	14	202	22.1	4.55	14	202	22.1	4.55	1.000
125	XP_015617463.1	uncharacterized protein LOC4327239 [Oryza sativa Japonica Group]	17	432	46.4	6.44	10	432	46.4	6.44	0.588
126	XP_015638049.1	embryonic abundant protein 1 [Oryza sativa Japonica Group]	19	95	10.2	5.60	17	95	10.2	5.60	0.895
127	XP_015642949.1	6-phosphogluconate dehydrogenase, decarboxylating 1 [Oryza sativa Japonica Group]	21	480	52.7	6.18	30	480	52.7	6.18	1.429
128	XP_015620584.1	pyruvate kinase 2, cytosolic [Oryza sativa Japonica Group]	17	527	57.4	6.76	18	527	57.4	6.76	1.059
129	BAA77337.1	Nad-dependent formate dehydrogenase [Oryza sativa]	23	376	41.2	7.34	19	376	41.2	7.34	0.826
130	XP_015628386.1	cysteine proteinase inhibitor 8 [Oryza sativa Japonica Group]	18	123	12.9	8.69	9	123	12.9	8.69	0.500
131	Q75H81.1	RecName: Full=Serpin-ZXA; AltName: Full=OrysaZxa	29	396	42.1	6.15	35	396	42.1	6.15	1.207
132	XP_015616831.1	pyruvate kinase 1, cytosolic [Oryza sativa Japonica Group]	17	527	57.3	6.76	18	527	57.3	6.76	1.059
133	BAB56043.1	lipoprotein-like [Oryza sativa Japonica Group]	20	252	28.0	7.93	22	252	28.0	7.93	1.100

134	BAD09607.1	putative superoxide dismutase [Cu-Zn], chloroplast precursor [Oryza sativa Japonica Group]	10	203	20.5	6.25	8	203	20.5	6.25	0.800
135	EAY87713.1	hypothetical protein OsI_09127 [Oryza sativa Indica Group]	20	635	68.2	5.34	19	635	68.2	5.34	0.950
136	AAX95683.1	hypothetical protein [Oryza sativa Japonica Group]	20	103	11.6	5.90	29	103	11.6	5.90	1.450
137	XP_015640657.1	60S acidic ribosomal protein P2B [Oryza sativa Japonica Group]	21	113	11.6	4.27	19	113	11.6	4.27	0.905
138	XP_015639563.1	uncharacterized protein LOC4339065 [Oryza sativa Japonica Group]	20	167	18.2	6.14	26	167	18.2	6.14	1.300
139	EAY87564.1	hypothetical protein OsI_08976 [Oryza sativa Indica Group]	20	314	35.5	7.55	19	314	35.5	7.55	0.950
140	BAA02253.1	elongation factor 1 beta' [Oryza sativa Japonica Group]	17	223	23.8	5.03	25	223	23.8	5.03	1.471
141	XP_015611712.1	glutathione transferase GST 23 [Oryza sativa Japonica Group]	18	223	25.2	5.71	15	223	25.2	5.71	0.833
142	XP_015628236.1	late embryogenesis abundant protein, group 3 [Oryza sativa Japonica Group]	18	400	41.8	8.38	42	400	41.8	8.38	2.333
143	XP_015625560.1	hsp70-Hsp90 organizing protein [Oryza sativa Japonica Group]	17	578	64.9	6.38	8	578	64.9	6.38	0.471
144	XP_015645043.1	reactive Intermediate Deaminase A, chloroplastic [Oryza sativa Japonica Group]	13	180	18.8	9.39	19	180	18.8	9.39	1.462
145	XP_015639765.1	guanosine nucleotide diphosphate dissociation inhibitor 2 [Oryza sativa Japonica Group]	20	445	49.7	5.73	12	445	49.7	5.73	0.600
146	EAY91599.1	hypothetical protein OsI_13234 [Oryza sativa Indica Group]	20	426	47.0	5.21	12	426	47.0	5.21	0.600
147	BAS79947.1	Os02g0637000, partial [Oryza sativa Japonica Group]	11	159	17.1	6.18	11	159	17.1	6.18	1.000
148	EAY85029.1	hypothetical protein OsI_06386 [Oryza sativa Indica Group]	19	878	98.3	5.17	11	878	98.3	5.17	0.579
149	XP_015618631.1	uncharacterized protein LOC4351498 [Oryza sativa Japonica Group]	20	147	16.4	5.59	16	147	16.4	5.59	0.800
150	XP_015646663.1	alpha-amylase inhibitor 5 [Oryza sativa Japonica Group]	18	154	15.9	6.52	17	154	15.9	6.52	0.944
151	XP_015625368.1	peptidyl-prolyl cis-trans isomerase FKBP12 [Oryza sativa Japonica Group]	12	112	12.1	7.96	10	112	12.1	7.96	0.833
152	AAO17017.1	Putative heat shock 70 KD protein, mitochondrial precursor [Oryza sativa Japonica Group]	20	656	70.4	5.58	25	656	70.4	5.58	1.250
153	XP_015646155.1	alpha-amylase/trypsin inhibitor [Oryza sativa Japonica Group]	12	148	15.8	7.50	19	148	15.8	7.50	1.583
154	BAA94761.1	adenylate kinase, partial [Oryza sativa]	12	209	22.8	8.06	9	209	22.8	8.06	0.750
155	BAS99345.1	Os06g0701100, partial [Oryza sativa Japonica Group]	14	400	45.6	5.67	1	400	45.6	5.67	0.071
156	BAD54224.1	putative 4-methyl-5(B-hydroxyethyl)-thiazol monophosphate biosynthesis enzyme [Oryza sativa Japonica Group]	17	394	41.7	5.66	23	394	41.7	5.66	1.353

157	EEC83720.1	hypothetical protein OsI_29556 [Oryza sativa Indica Group]	15	271	30.5	8.02	13	271	30.5	8.02	0.867
158	XP_015651405.1	peptidyl-prolyl cis-trans isomerase FKBP15-1 [Oryza sativa Japonica Group]	13	154	16.3	5.38	12	154	16.3	5.38	0.923
159	AAG28777.1	citrate synthase [Oryza sativa]	10	472	52.2	7.88	4	472	52.2	7.88	0.400
160	XP_015617060.1	translationally-controlled tumor protein homolog [Oryza sativa Japonica Group]	13	168	18.9	4.68	15	168	18.9	4.68	1.154
161	XP_015612407.1	cytosolic isocitrate dehydrogenase [NADP] [Oryza sativa Japonica Group]	15	412	46.0	6.80	11	412	46.0	6.80	0.733
162	AAF73828.1	aldehyde dehydrogenase [Oryza sativa]	12	549	59.3	6.80	6	549	59.3	6.80	0.500
163	XP_015617608.1	water stress-inducible protein Rab21 [Oryza sativa Japonica Group]	15	172	17.3	9.20	16	172	17.3	9.20	1.067
164	XP_015631105.1	pathogenesis-related protein 1 [Oryza sativa Japonica Group]	13	160	17.2	6.28	9	160	17.2	6.28	0.692
165	XP_015627830.1	ran-binding protein 1 homolog a [Oryza sativa Japonica Group]	9	209	23.4	4.79	10	209	23.4	4.79	1.111
166	XP_015631880.1	ricin B-like lectin R40C1 [Oryza sativa Japonica Group]	14	348	38.8	6.80	9	348	38.8	6.80	0.643
167	EAZ07074.1	hypothetical protein OsI_29320 [Oryza sativa Indica Group]	14	256	28.8	4.84	14	256	28.8	4.84	1.000
168	BAT14238.1	Os11g0525600, partial [Oryza sativa Japonica Group]	12	904	101.4	6.24	14	904	101.4	6.24	1.167
169	XP_015643571.1	early nodulin-like protein 1 [Oryza sativa Japonica Group]	13	237	24.9	5.07	8	237	24.9	5.07	0.615
170	AAK16176.1	translation initiation factor 5A [Oryza sativa Japonica Group]	17	161	17.5	6.14	11	161	17.5	6.14	0.647
171	EAZ12194.1	hypothetical protein OsJ_02079 [Oryza sativa Japonica Group]	14	243	26.1	5.57	11	243	26.1	5.57	0.786
172	BAS95819.1	Os06g0114000, partial [Oryza sativa Japonica Group]	16	520	55.5	5.06	2	520	55.5	5.06	0.125
173	BAT16466.1	Os12g0235800, partial [Oryza sativa Japonica Group]	17	350	38.9	6.21	11	350	38.9	6.21	0.647
174	XP_015633442.1	protein DJ-1 homolog D isoform X2 [Oryza sativa Japonica Group]	14	387	41.3	5.54	7	387	41.3	5.54	0.500
175	XP_015644758.1	40S ribosomal protein S12 [Oryza sativa Japonica Group]	14	138	14.8	5.52	14	138	14.8	5.52	1.000
176	AAG32472.1	putative glutathione S-transferase OsGSTU3 [Oryza sativa Japonica Group]	17	233	25.3	6.00	16	233	25.3	6.00	0.941
177	AAT01337.1	putative nascent polypeptide associated complex alpha chain [Oryza sativa Japonica Group]	7	128	13.7	4.98	8	128	13.7	4.98	1.143
178	XP_015644252.1	PLAT domain-containing protein 3 [Oryza sativa Japonica Group]	11	171	18.1	6.65	4	171	18.1	6.65	0.364
179	XP_015618529.1	GEM-like protein 5 [Oryza sativa Japonica Group]	17	298	31.5	6.80	11	298	31.5	6.80	0.647
180	BAF29005.1	Os12g0115000, partial [Oryza sativa Japonica Group]	14	110	10.7	8.34	14	110	10.7	8.34	1.000

181	EEC81650.1	hypothetical protein OsI_25188 [Oryza sativa Indica Group]	14	533	57.1	6.35	21	533	57.1	6.35	1.500
182	XP_015648059.1	enoyl-[acyl-carrier-protein] reductase [NADH] 1, chloroplastic [Oryza sativa Japonica Group]	12	375	39.1	8.68	11	375	39.1	8.68	0.917
183	XP_025881413.1	chaperone protein ClpB1 isoform X2 [Oryza sativa Japonica Group]	9	874	96.2	6.16	4	874	96.2	6.16	0.444
184	XP_015625080.1	late embryogenesis abundant protein Lea14-A [Oryza sativa Japonica Group]	11	151	16.2	5.17	10	151	16.2	5.17	0.909
185	AAO37499.1	expressed protein [Oryza sativa Japonica Group]	11	158	16.5	4.46	7	158	16.5	4.46	0.636
186	EEE53911.1	hypothetical protein OsJ_00464 [Oryza sativa Japonica Group]	11	93	10.1	6.84	26	93	10.1	6.84	2.364
187	XP_015617609.1	dehydrin Rab16B [Oryza sativa Japonica Group]	6	164	16.5	9.28	9	164	16.5	9.28	1.500
188	XP_015611441.1	uncharacterized protein LOC4346534 [Oryza sativa Japonica Group]	11	95	10.3	4.81	2	95	10.3	4.81	0.182
189	EAZ00429.1	hypothetical protein OsI_22453 [Oryza sativa Indica Group]	10	413	43.1	4.77	10	413	43.1	4.77	1.000
190	ABL98203.1	isocitrate lyase [Oryza sativa Japonica Group]	8	562	62.0	7.23	1	562	62.0	7.23	0.125
191	XP_015627998.1	uncharacterized protein LOC4333625 [Oryza sativa Japonica Group]	9	141	15.2	5.80	11	141	15.2	5.80	1.222
192	EEE64920.1	hypothetical protein OsJ_19780 [Oryza sativa Japonica Group]	13	239	26.0	8.75	8	239	26.0	8.75	0.615
193	EAY76362.1	hypothetical protein OsI_04294 [Oryza sativa Indica Group]	16	396	41.7	7.77	21	396	41.7	7.77	1.313
194	EEE53627.1	hypothetical protein OsJ_36903 [Oryza sativa Japonica Group]	8	271	28.3	4.93	9	271	28.3	4.93	1.125
195	BAA03711.1	brain specific protein [Oryza sativa]	15	260	29.1	4.83	20	260	29.1	4.83	1.333
196	A2WNH1.2	RecName: Full=Calmodulin-3; Short=CaM-3	8	149	16.8	4.27	15	149	16.8	4.27	1.875
197	XP_015629251.1	oleosin 18 kDa [Oryza sativa Japonica Group]	9	172	17.2	10.10	10	172	17.2	10.10	1.111
198	BAD88157.1	protein kinase-like [Oryza sativa Japonica Group]	11	463	49.6	7.97	8	463	49.6	7.97	0.727
199	AAO72574.1	elongation factor 1 gamma-like protein, partial [Oryza sativa Japonica Group]	7	409	46.5	6.61	11	409	46.5	6.61	1.571
200	XP_015635902.1	malate synthase [Oryza sativa Japonica Group]	12	567	62.6	7.14	8	567	62.6	7.14	0.667
201	XP_015627232.1	60S ribosomal protein L12-1 [Oryza sativa Japonica Group]	11	166	17.7	9.16	16	166	17.7	9.16	1.455
202	EAY72790.1	hypothetical protein OsI_00654 [Oryza sativa Indica Group]	11	813	89.7	5.14	11	813	89.7	5.14	1.000
203	EEC78534.1	hypothetical protein OsI_18485 [Oryza sativa Indica Group]	6	106	10.0	8.91	4	106	10.0	8.91	0.667
204	XP_015635492.1	probable inactive UDP-arabinopyranose mutase 2 [Oryza sativa Japonica Group]	12	347	38.9	6.44	6	347	38.9	6.44	0.500

205	XP_015611390.1	uncharacterized protein LOC4346597 [Oryza sativa Japonica Group]	9	185	17.3	7.65	6	185	17.3	7.65	0.667
206	XP_015640756.1	photosynthetic NDH subunit of lumenal location 5, chloroplastic [Oryza sativa Japonica Group]	7	250	26.6	9.29	7	250	26.6	9.29	1.000
207	AAN05517.1	unknown protein [Oryza sativa Japonica Group]	12	317	35.4	5.87	22	317	35.4	5.87	1.833
208	XP_015649943.1	aminopeptidase M1-B [Oryza sativa Japonica Group]	13	875	97.8	5.39	10	875	97.8	5.39	0.769
209	XP_015633089.1	peptide methionine sulfoxide reductase B5 [Oryza sativa Japonica Group]	12	136	14.7	7.24	13	136	14.7	7.24	1.083
210	AAO23563.1	aspartate aminotransferase, partial [Oryza sativa]	11	414	45.8	6.28	5	414	45.8	6.28	0.455
211	ABR25841.1	40S ribosomal protein S8, partial [Oryza sativa Indica Group]	14	168	18.8	9.69	11	168	18.8	9.69	0.786
212	AGT38452.1	phosphoglucose isomerase [Oryza sativa Japonica Group]	13	567	62.5	7.28	14	567	62.5	7.28	1.077
213	XP_015650850.1	nuclear transport factor 2 [Oryza sativa Japonica Group]	5	122	13.3	5.69	3	122	13.3	5.69	0.600
214	XP_015617770.1	uncharacterized protein DDB_G0286299 [Oryza sativa Japonica Group]	9	331	35.3	4.98	17	331	35.3	4.98	1.889
215	XP_015637493.1	oil body-associated protein 1A [Oryza sativa Japonica Group]	15	238	26.4	5.96	14	238	26.4	5.96	0.933
216	AIC77182.1	lipid transfer protein 2 [Oryza sativa]	11	95	9.4	8.95	19	95	9.4	8.95	1.727
217	XP_006654825.1	PREDICTED: GTP-binding nuclear protein Ran-2 [Oryza brachyantha]	15	221	25.0	7.12	17	221	25.0	7.12	1.133
218	EAY79716.1	hypothetical protein OsI_34868 [Oryza sativa Indica Group]	7	118	11.5	9.07	5	118	11.5	9.07	0.714
219	CAG28936.1	lipid transfer protein [Oryza sativa Japonica Group]	13	110	11.2	8.69	10	110	11.2	8.69	0.769
220	XP_015627797.1	hydroxyacylglutathione hydrolase cytoplasmic [Oryza sativa Japonica Group]	9	258	28.5	5.64	11	258	28.5	5.64	1.222
221	EEC74290.1	hypothetical protein OsI_09541 [Oryza sativa Indica Group]	6	413	46.1	6.04	1	413	46.1	6.04	0.167
222	XP_015633704.1	23.2 kDa heat shock protein [Oryza sativa Japonica Group]	7	215	23.2	5.54	15	215	23.2	5.54	2.143
223	AAX95269.1	glucan endo-1,3-beta-glucosidase precursor (ec 3.2.1.39) ((1-3)-beta-glucan endohydrolase) ((1-3)-beta-glucanase) (beta-1,3-endoglucanase) [Oryza sativa Japonica Group]	6	431	45.9	6.44	3	431	45.9	6.44	0.500
224	EEC77777.1	hypothetical protein OsI_16938 [Oryza sativa Indica Group]	8	168	18.5	7.81	19	168	18.5	7.81	2.375
225	BAD87551.1	putative fructokinase [Oryza sativa Japonica Group]	8	245	27.2	5.29	7	245	27.2	5.29	0.875
226	XP_006644130.1	PREDICTED: ubiquitin-40S ribosomal protein S27a-1 [Oryza brachyantha]	13	155	17.7	9.77	16	155	17.7	9.77	1.231
227	XP_015626829.1	cyclase-like protein 1 [Oryza sativa Japonica Group]	10	274	29.4	6.68	15	274	29.4	6.68	1.500

228	BAD81175.1	putative cysteine proteinase inhibitor [Oryza sativa Japonica Group]	8	208	23.3	6.23	5	208	23.3	6.23	0.625
229	ABR25339.1	giberellin regulated family protein, partial [Oryza sativa Indica Group]	6	61	6.6	8.43	4	61	6.6	8.43	0.667
230	BAF17027.1	Os05g0295800 [Oryza sativa Japonica Group]	12	189	21.3	6.23	6	189	21.3	6.23	0.500
231	XP_015650763.1	fasciclin-like arabinogalactan protein 1 [Oryza sativa Japonica Group]	7	415	43.6	8.24	4	415	43.6	8.24	0.571
232	BAC06273.1	putative 60S ribosomal protein L5 [Oryza sativa Japonica Group]	10	301	34.3	9.09	5	301	34.3	9.09	0.500
233	BAA77214.1	cytosolic monodehydroascorbate reductase [Oryza sativa Japonica Group]	9	435	46.6	5.71	13	435	46.6	5.71	1.444
234	BAG90611.1	unnamed protein product [Oryza sativa Japonica Group]	6	221	24.9	6.67	1	221	24.9	6.67	0.167
235	XP_015627045.1	protein disulfide isomerase-like 1-4 [Oryza sativa Japonica Group]	10	563	62.2	4.93	10	563	62.2	4.93	1.000
236	ABR25392.1	Bowman-Birk type bran trypsin inhibitor, partial [Oryza sativa Indica Group]	13	217	24.2	5.78	25	217	24.2	5.78	1.923
237	AAD27887.1	lectin, partial [Oryza sativa Japonica Group]	7	200	20.2	5.40	8	200	20.2	5.40	1.143
238	3FR7_A	Chain A, Ketol-Acid Reductoisomerase (Kari) In Complex With Mg2+	9	525	57.3	5.67	13	525	57.3	5.67	1.444
239	XP_015632349.1	40S ribosomal protein S3-2 [Oryza sativa Japonica Group]	6	228	25.4	9.52	12	228	25.4	9.52	2.000
240	XP_015648168.1	acyl-CoA-binding domain-containing protein 1-like [Oryza sativa Japonica Group]	8	91	10.1	5.22	10	91	10.1	5.22	1.250
241	XP_015610625.1	peptidyl-prolyl cis-trans isomerase CYP19-3 [Oryza sativa Japonica Group]	5	179	19.2	7.78	3	179	19.2	7.78	0.600
242	EAY92778.1	hypothetical protein OsI_14582 [Oryza sativa Indica Group]	13	703	80.2	5.17	16	703	80.2	5.17	1.231
243	XP_015630351.1	proteasome subunit alpha type-6 isoform X2 [Oryza sativa Japonica Group]	11	246	27.6	6.65	6	246	27.6	6.65	0.545
244	EEC83682.1	hypothetical protein OsI_29480 [Oryza sativa Indica Group]	5	226	23.3	8.10	1	226	23.3	8.10	0.200
245	EAY97646.1	hypothetical protein OsI_19569 [Oryza sativa Indica Group]	10	393	43.2	7.08	6	393	43.2	7.08	0.600
246	XP_015647771.1	superoxide dismutase [Cu-Zn] 2 [Oryza sativa Japonica Group]	7	152	15.1	6.42	10	152	15.1	6.42	1.429
247	CAE03380.1	OSJNBa0004N05.4 [Oryza sativa Japonica Group]	8	243	28.0	5.87	11	243	28.0	5.87	1.375
248	EAZ09670.1	hypothetical protein OsI_31953 [Oryza sativa Indica Group]	9	178	19.6	8.22	10	178	19.6	8.22	1.111
249	CAH67861.1	B0403H10-OSIGBa0105A11.13 [Oryza sativa]	7	110	12.3	8.50	9	110	12.3	8.50	1.286
250	XP_015612568.1	uncharacterized protein LOC4346387 [Oryza sativa Japonica Group]	9	75	7.8	5.86	12	75	7.8	5.86	1.333

251	EAZ38239.1	hypothetical protein OsJ_22614 [Oryza sativa Japonica Group]	7	204	21.7	9.26	17	204	21.7	9.26	2.429
252	EEC70832.1	hypothetical protein OsI_02319 [Oryza sativa Indica Group]	6	314	33.3	5.40	10	314	33.3	5.40	1.667
253	XP_015650416.1	uncharacterized protein LOC4344438 [Oryza sativa Japonica Group]	5	96	10.5	5.26	10	96	10.5	5.26	2.000
254	XP_015644062.1	T-complex protein 1 subunit gamma [Oryza sativa Japonica Group]	6	558	60.9	6.65	6	558	60.9	6.65	1.000
255	AAM13448.1	chalcone isomerase [Oryza sativa Japonica Group]	7	233	23.8	5.27	7	233	23.8	5.27	1.000
256	BAS86779.1	Os03g0790900, partial [Oryza sativa Japonica Group]	6	1120	120.5	6.19	6	1120	120.5	6.19	1.000
257	XP_015625812.1	succinate--CoA ligase [ADP-forming] subunit beta, mitochondrial [Oryza sativa Japonica Group]	7	422	45.1	6.32	3	422	45.1	6.32	0.429
258	EAZ11740.1	hypothetical protein OsJ_01605 [Oryza sativa Japonica Group]	9	467	49.8	6.28	5	467	49.8	6.28	0.556
259	EAZ16056.1	hypothetical protein OsJ_31497 [Oryza sativa Japonica Group]	8	128	14.9	9.92	2	128	14.9	9.92	0.250
260	XP_015649763.1	tricin synthase 2 isoform X2 [Oryza sativa Japonica Group]	8	292	32.0	5.71	5	292	32.0	5.71	0.625
261	XP_015635772.1	pyruvate kinase, cytosolic isozyme [Oryza sativa Japonica Group]	11	511	55.2	7.91	18	511	55.2	7.91	1.636
262	XP_015618089.1	probable aspartyl aminopeptidase [Oryza sativa Japonica Group]	8	478	52.4	6.92	7	478	52.4	6.92	0.875
263	XP_015640796.1	thioredoxin H-type [Oryza sativa Japonica Group]	10	121	13.2	5.08	7	121	13.2	5.08	0.700
264	BAT07058.1	Os09g0252100, partial [Oryza sativa Japonica Group]	6	389	39.7	6.90	11	389	39.7	6.90	1.833
265	XP_015643271.1	DNA-binding protein MNB1B [Oryza sativa Japonica Group]	6	157	17.1	5.95	5	157	17.1	5.95	0.833
266	AAT85124.1	putative 60s ribosomal protein L15 [Oryza sativa Japonica Group]	6	186	22.1	11.68	6	186	22.1	11.68	1.000
267	XP_015650487.1	uncharacterized protein LOC4344973 isoform X3 [Oryza sativa Japonica Group]	11	216	23.4	9.64	3	216	23.4	9.64	0.273
268	EAY84650.1	hypothetical protein OsI_06022 [Oryza sativa Indica Group]	3	156	16.8	4.97	2	156	16.8	4.97	0.667
269	XP_015650041.1	60S acidic ribosomal protein P0 [Oryza sativa Japonica Group]	9	319	34.4	5.49	6	319	34.4	5.49	0.667
270	BAF29008.2	Os12g0115500, partial [Oryza sativa Japonica Group]	6	93	9.5	9.00	2	93	9.5	9.00	0.333
271	XP_015618339.1	60S acidic ribosomal protein P3-like [Oryza sativa Japonica Group]	8	119	11.9	4.41	5	119	11.9	4.41	0.625
272	XP_015619737.1	uncharacterized protein LOC9267984 [Oryza sativa Japonica Group]	6	164	16.7	8.05	8	164	16.7	8.05	1.333
273	XP_015635666.1	cysteine proteinase inhibitor 10 [Oryza sativa Japonica Group]	7	151	15.4	10.15	4	151	15.4	10.15	0.571

274	XP_015639713.1	actin-1 [Oryza sativa Japonica Group]	8	376	41.6	5.48	4	376	41.6	5.48	0.500
275	ABF94326.1	40S ribosomal protein SA, putative, expressed [Oryza sativa Japonica Group]	10	269	29.8	5.48	6	269	29.8	5.48	0.600
276	XP_025883358.1	copper transport protein ATX1 isoform X2 [Oryza sativa Japonica Group]	7	79	8.3	7.96	3	79	8.3	7.96	0.429
277	XP_015643961.1	prolamin PPROL 17D-like [Oryza sativa Japonica Group]	8	149	16.7	7.90	9	149	16.7	7.90	1.125
278	XP_015621051.1	cysteine proteinase inhibitor 5 [Oryza sativa Japonica Group]	5	148	15.7	6.13	3	148	15.7	6.13	0.600
279	XP_015643710.1	uncharacterized protein At5g39570 [Oryza sativa Japonica Group]	4	381	41.6	5.16	5	381	41.6	5.16	1.250
280	EAY86398.1	hypothetical protein OsI_07776 [Oryza sativa Indica Group]	7	378	40.3	9.25	5	378	40.3	9.25	0.714
281	AAF61489.1	UMP synthase, partial [Oryza sativa Japonica Group]	5	316	33.8	7.93	3	316	33.8	7.93	0.600
282	AAW34237.1	putative ribosomal protein S19 [Oryza sativa Japonica Group]	12	144	16.2	9.94	7	144	16.2	9.94	0.583
283	XP_015648122.1	uncharacterized protein LOC4345453 [Oryza sativa Japonica Group]	4	91	9.1	5.01	2	91	9.1	5.01	0.500
284	AAL40390.1	C13 cysteine proteinase precursor [Oryza sativa Indica Group]	8	465	51.9	6.04	12	465	51.9	6.04	1.500
285	AXE15814.1	phospholipase D alpha1, partial [Oryza glaberrima]	5	744	84.6	6.19	5	744	84.6	6.19	1.000
286	XP_015650124.1	glutamate decarboxylase 1 isoform X2 [Oryza sativa Japonica Group]	6	497	56.2	5.60	8	497	56.2	5.60	1.333
287	XP_015622682.1	fumarylacetoacetase-like [Oryza sativa Japonica Group]	6	429	47.1	5.94	10	429	47.1	5.94	1.667
288	EEC82655.1	hypothetical protein OsI_27268 [Oryza sativa Indica Group]	4	478	53.3	5.66	5	478	53.3	5.66	1.250
289	EAY99887.1	hypothetical protein OsI_21882 [Oryza sativa Indica Group]	6	226	24.1	6.54	4	226	24.1	6.54	0.667
290	YP_588368.1	ATPase subunit 9 (mitochondrion) [Zea mays subsp. mays]	2	74	7.6	8.75	3	74	7.6	8.75	1.500
291	XP_015632320.1	uncharacterized protein LOC4334013 [Oryza sativa Japonica Group]	6	82	8.6	5.15	4	82	8.6	5.15	0.667
292	BAS81339.1	Os02g0793700, partial [Oryza sativa Japonica Group]	7	101	10.7	6.74	2	101	10.7	6.74	0.286
293	BAS75388.1	Os01g0866600, partial [Oryza sativa Japonica Group]	3	73	7.8	6.77	5	73	7.8	6.77	1.667
294	XP_015640375.1	protein disulfide isomerase-like 2-1 isoform X2 [Oryza sativa Japonica Group]	10	366	39.9	6.99	8	366	39.9	6.99	0.800
295	XP_015625595.1	peptidyl-prolyl cis-trans isomerase FKBP15-2 isoform X2 [Oryza sativa Japonica Group]	6	137	14.3	9.50	6	137	14.3	9.50	1.000
296	BAD08003.1	putative Proteasome subunit alpha type 1 [Oryza sativa Japonica Group]	6	245	26.6	5.68	5	245	26.6	5.68	0.833
297	ABR26039.1	cbs domain protein, partial [Oryza sativa Indica Group]	6	141	15.6	8.15	3	141	15.6	8.15	0.500

298	EAY86982.1	hypothetical protein OsI_08376 [Oryza sativa Indica Group]	4	238	25.8	9.23	6	238	25.8	9.23	1.500
299	XP_015630177.1	uncharacterized protein LOC4331521 [Oryza sativa Japonica Group]	7	115	12.6	5.33	11	115	12.6	5.33	1.571
300	XP_015631704.1	thioredoxin H2-2 [Oryza sativa Japonica Group]	7	134	14.8	5.97	5	134	14.8	5.97	0.714
301	XP_015619738.1	osmotin-like protein [Oryza sativa Japonica Group]	6	233	23.4	8.02	6	233	23.4	8.02	1.000
302	ABF98824.1	Stem-specific protein TSJT1, putative, expressed [Oryza sativa Japonica Group]	5	194	21.3	6.38	1	194	21.3	6.38	0.200
303	BAS71712.1	Os01g0300200, partial [Oryza sativa Japonica Group]	4	487	52.9	8.10	8	487	52.9	8.10	2.000
304	XP_015647977.1	MD-2-related lipid-recognition protein ROSY1 [Oryza sativa Japonica Group]	5	152	16.2	5.50	4	152	16.2	5.50	0.800
305	XP_015638298.1	gibberellin-regulated protein 2 [Oryza sativa Japonica Group]	5	92	9.7	8.44	6	92	9.7	8.44	1.200
306	BAD09646.1	putative lipid transfer protein precursor [Oryza sativa Japonica Group]	5	120	12.0	7.84	3	120	12.0	7.84	0.600
307	XP_015614155.1	probable aquaporin TIP3-1 [Oryza sativa Japonica Group]	4	264	27.5	8.50	10	264	27.5	8.50	2.500
308	AAA33917.1	superoxide dismutase [Oryza sativa Japonica Group]	6	152	15.2	6.18	7	152	15.2	6.18	1.167
309	XP_015622490.1	60S ribosomal protein L23A [Oryza sativa Japonica Group]	7	152	17.0	10.30	7	152	17.0	10.30	1.000
310	BAS96625.1	Os06g0197550 [Oryza sativa Japonica Group]	4	119	13.3	4.73	1	119	13.3	4.73	0.250
311	XP_015621006.1	ricin B-like lectin R40G3 [Oryza sativa Japonica Group]	4	268	30.1	6.77	2	268	30.1	6.77	0.500
312	XP_015642483.1	profilin LP04 [Oryza sativa Japonica Group]	3	131	14.1	4.87	1	131	14.1	4.87	0.333
313	BAD28950.1	putative glutathione transferase [Oryza sativa Japonica Group]	6	328	37.4	6.30	2	328	37.4	6.30	0.333
314	XP_015622836.1	outer envelope membrane protein 7 [Oryza sativa Japonica Group]	4	101	10.4	4.55	1	101	10.4	4.55	0.250
315	XP_015644653.1	polygalacturonase inhibitor 1 [Oryza sativa Japonica Group]	5	332	35.4	7.42	4	332	35.4	7.42	0.800
316	EEE57671.1	hypothetical protein OsJ_08115 [Oryza sativa Japonica Group]	5	892	98.1	5.59	7	892	98.1	5.59	1.400
317	BAD73062.1	putative 4-methyl-5(B-hydroxyethyl)-thiazol monophosphate biosynthesis enzyme [Oryza sativa Japonica Group]	6	426	45.1	6.30	4	426	45.1	6.30	0.667
318	BAF20971.1	Os07g0184300 [Oryza sativa Japonica Group]	5	118	13.4	10.73	7	118	13.4	10.73	1.400
319	BAF06555.1	Os01g0819500, partial [Oryza sativa Japonica Group]	4	47	5.4	5.87	1	47	5.4	5.87	0.250
320	BAA90487.1	heat shock protein 90 [Oryza sativa]	4	810	92.7	4.98	10	810	92.7	4.98	2.500
321	XP_015620382.1	major pollen allergen Bet v 1-D/H [Oryza sativa Japonica Group]	5	158	16.7	5.00	5	158	16.7	5.00	1.000
322	XP_015630764.1	alcohol dehydrogenase-like 2 [Oryza sativa Japonica Group]	6	379	40.3	5.29	4	379	40.3	5.29	0.667

323	EAY94706.1	hypothetical protein OsI_16484 [Oryza sativa Indica Group]	6	345	37.6	6.73	2	345	37.6	6.73	0.333
324	XP_015639036.1	programmed cell death protein 5 [Oryza sativa Japonica Group]	5	128	14.5	6.60	6	128	14.5	6.60	1.200
325	EAZ18268.1	hypothetical protein OsJ_33805 [Oryza sativa Japonica Group]	6	447	49.0	7.46	3	447	49.0	7.46	0.500
326	ABA96281.1	Nonspecific lipid-transfer protein 5 precursor, putative, expressed [Oryza sativa Japonica Group]	4	106	10.6	8.13	7	106	10.6	8.13	1.750
327	XP_015630123.1	late embryogenesis abundant protein 47 [Oryza sativa Japonica Group]	4	214	21.3	4.56	7	214	21.3	4.56	1.750
328	XP_015624320.2	ribulose bisphosphate carboxylase small chain clone 512 [Oryza sativa Japonica Group]	5	183	20.6	8.73	6	183	20.6	8.73	1.200
329	XP_015625225.1	glycine-rich protein 2 [Oryza sativa Japonica Group]	3	241	22.7	7.06	9	241	22.7	7.06	3.000
330	BAD33762.1	putative 6-phosphogluconolactonase [Oryza sativa Japonica Group]	5	269	29.0	5.71	3	269	29.0	5.71	0.600
331	CAI29542.1	eukaryotic translation initiation factor 2 alpha [Oryza sativa Indica Group]	4	339	38.2	5.40	3	339	38.2	5.40	0.750
332	AEZ03740.1	hypothetical protein (mitochondrion) [Oryza sativa Indica Group]	6	389	43.6	7.28	5	389	43.6	7.28	0.833
333	BAA96837.1	beta 4 subunit of 20S proteasome [Oryza sativa Japonica Group]	4	212	23.4	5.64	3	212	23.4	5.64	0.750
334	XP_015610948.1	oleosin 16 kDa-like [Oryza sativa Japonica Group]	2	158	15.6	6.80	2	158	15.6	6.80	1.000
335	XP_015622053.1	glycine-rich RNA-binding protein 4, mitochondrial [Oryza sativa Japonica Group]	3	150	15.5	8.44	1	150	15.5	8.44	0.333
336	AAF70196.1	putative histone deacetylase HD2 [Oryza sativa]	4	297	32.5	4.70	4	297	32.5	4.70	1.000
337	EEE60037.1	hypothetical protein OsJ_12809 [Oryza sativa Japonica Group]	5	437	45.9	7.01	3	437	45.9	7.01	0.600
338	EEC73763.1	hypothetical protein OsI_08429 [Oryza sativa Indica Group]	2	1066	118.6	9.42	2	1066	118.6	9.42	1.000
339	XP_015630536.1	cytochrome c oxidase subunit 6b-1 [Oryza sativa Japonica Group]	4	169	18.9	4.30	3	169	18.9	4.30	0.750
340	XP_015617601.1	40S ribosomal protein S5 [Oryza sativa Japonica Group]	7	199	22.2	9.70	3	199	22.2	9.70	0.429
341	BAS93533.1	Os05g0350600, partial [Oryza sativa Japonica Group]	5	77	8.3	4.59	7	77	8.3	4.59	1.400
342	XP_015621389.1	Bowman-Birk type bran trypsin inhibitor [Oryza sativa Japonica Group]	5	179	19.3	7.75	8	179	19.3	7.75	1.600
343	XP_015633122.1	galactokinase [Oryza sativa Japonica Group]	5	506	54.7	6.89	2	506	54.7	6.89	0.400
344	XP_015642917.1	GDSL esterase/lipase At4g01130 [Oryza sativa Japonica Group]	4	379	40.9	8.15	11	379	40.9	8.15	2.750
345	EAY94163.1	hypothetical protein OsI_15938 [Oryza sativa Indica Group]	4	288	31.3	7.61	1	288	31.3	7.61	0.250

346	XP_006652294.1	PREDICTED: 40S ribosomal protein S10 [Oryza brachyantha]	5	183	20.3	9.76	3	183	20.3	9.76	0.600
347	1EQK_A	Chain A, Solution Structure Of Oryzacystatin-I, A Cysteine Proteinase Inhibitor Of The Rice, Oryza Sativa L. Japonica	5	102	11.4	5.06	9	102	11.4	5.06	1.800
348	EAY87625.1	hypothetical protein OsI_09036 [Oryza sativa Indica Group]	5	168	17.8	6.54	9	168	17.8	6.54	1.800
349	XP_015628927.1	uncharacterized protein LOC4332927 [Oryza sativa Japonica Group]	5	114	12.7	7.81	8	114	12.7	7.81	1.600
350	ABF99927.1	Elongation factor Tu, mitochondrial precursor, putative, expressed [Oryza sativa Japonica Group]	4	318	33.9	5.96	2	318	33.9	5.96	0.500
351	EAY72729.1	hypothetical protein OsI_00594 [Oryza sativa Indica Group]	4	124	13.2	9.63	3	124	13.2	9.63	0.750
352	EEC79555.1	hypothetical protein OsI_20679 [Oryza sativa Indica Group]	2	567	62.4	6.83	8	567	62.4	6.83	4.000
353	WP_129325415.1	hypothetical protein [Klebsiella pneumoniae]	3	98	10.6	5.01	1	98	10.6	5.01	0.333
354	BAA02157.1	40S subunit ribosomal protein [Oryza sativa Japonica Group]	3	117	13.0	9.63	1	117	13.0	9.63	0.333
355	EAZ29242.1	hypothetical protein OsJ_13305 [Oryza sativa Japonica Group]	5	688	73.6	5.74	7	688	73.6	5.74	1.400
356	EEC75442.1	hypothetical protein OsI_11975 [Oryza sativa Indica Group]	5	581	64.4	4.93	3	581	64.4	4.93	0.600
357	BAD27731.1	phosphoenolpyruvate carboxylase-like [Oryza sativa Japonica Group]	5	452	51.7	5.80	2	452	51.7	5.80	0.400
358	CAD41669.3	OSJNBa0019K04.16 [Oryza sativa Japonica Group]	4	431	44.5	6.65	16	431	44.5	6.65	4.000
359	XP_015629015.1	PLASMODESMATA CALLOSE-BINDING PROTEIN 2 [Oryza sativa Japonica Group]	4	175	17.8	4.83	3	175	17.8	4.83	0.750
360	EEE61517.1	hypothetical protein OsJ_15810 [Oryza sativa Japonica Group]	2	430	45.2	10.21	4	430	45.2	10.21	2.000
361	ABA99939.2	Malate dehydrogenase, glyoxysomal precursor, putative, expressed [Oryza sativa Japonica Group]	5	320	33.6	7.62	1	320	33.6	7.62	0.200
362	EEC72589.1	hypothetical protein OsI_06045 [Oryza sativa Indica Group]	6	527	56.0	8.10	10	527	56.0	8.10	1.667
363	XP_015615828.1	PITH domain-containing protein 1 isoform X1 [Oryza sativa Japonica Group]	4	204	22.6	5.06	2	204	22.6	5.06	0.500
364	CAE05728.2	OSJNBb0017I01.8 [Oryza sativa Japonica Group]	3	152	16.3	7.84	2	152	16.3	7.84	0.667
365	BAS95716.1	Os06g0103300, partial [Oryza sativa Japonica Group]	4	328	36.2	6.02	1	328	36.2	6.02	0.250
366	XP_015641790.1	pyruvate dehydrogenase E1 component subunit alpha-2, mitochondrial [Oryza sativa Japonica Group]	4	398	43.6	8.59	1	398	43.6	8.59	0.250
367	ABR25651.1	regulator of ribonuclease activity a, partial [Oryza sativa Indica Group]	4	167	17.9	5.88	7	167	17.9	5.88	1.750

392	EEC82447.1	hypothetical protein OsI_26881 [Oryza sativa Indica Group]	3	546	59.1	5.90	1	546	59.1	5.90	0.333
393	XP_015644407.1	eukaryotic translation initiation factor 1A [Oryza sativa Japonica Group]	3	144	16.3	5.35	1	144	16.3	5.35	0.333
394	XP_015622112.1	60S ribosomal protein L30 [Oryza sativa Japonica Group]	2	111	12.4	9.58	1	111	12.4	9.58	0.500
395	AAA70046.1	lipid transfer protein precursor, partial [Oryza sativa]	2	99	10.0	8.51	5	99	10.0	8.51	2.500
396	EAY88614.1	hypothetical protein OsI_10089 [Oryza sativa Indica Group]	3	424	44.8	8.29	7	424	44.8	8.29	2.333
397	XP_006658032.1	PREDICTED: 10 kDa chaperonin-like [Oryza brachyantha]	4	98	10.6	8.38	1	98	10.6	8.38	0.250
398	EEE58288.1	hypothetical protein OsJ_09326 [Oryza sativa Japonica Group]	3	907	98.8	6.16	3	907	98.8	6.16	1.000
399	BAH93547.1	Os06g0515301 [Oryza sativa Japonica Group]	3	104	11.3	8.40	1	104	11.3	8.40	0.333
400	XP_015626163.1	4-hydroxyphenylpyruvate dioxygenase [Oryza sativa Japonica Group]	2	446	46.9	5.55	1	446	46.9	5.55	0.500
401	EEE56720.1	hypothetical protein OsJ_06222 [Oryza sativa Japonica Group]	4	273	30.1	9.06	1	273	30.1	9.06	0.250
402	XP_015630795.1	purple acid phosphatase 3 [Oryza sativa Japonica Group]	3	339	38.3	7.59	3	339	38.3	7.59	1.000
403	EAY98550.1	hypothetical protein OsI_20462 [Oryza sativa Indica Group]	2	132	14.2	4.61	6	132	14.2	4.61	3.000
404	BAD19822.1	hypothetical protein [Oryza sativa Japonica Group]	3	81	8.4	5.86	2	81	8.4	5.86	0.667
405	XP_015636840.1	dihydrolipoyllysine-residue succinyltransferase component of 2-oxoglutarate dehydrogenase complex 1, mitochondrial isoform X2 [Oryza sativa Japonica Group]	2	439	48.1	8.38	2	439	48.1	8.38	1.000
406	ABF99925.1	NAC domain containing protein, expressed [Oryza sativa Japonica Group]	2	146	16.1	6.01	2	146	16.1	6.01	1.000
407	EEC79938.1	hypothetical protein OsI_21522 [Oryza sativa Indica Group]	4	401	44.2	5.80	2	401	44.2	5.80	0.500
408	XP_015632687.1	subtilisin-like protease SBT1.7 [Oryza sativa Japonica Group]	4	764	78.5	6.67	5	764	78.5	6.67	1.250
409	XP_015629722.1	non-specific lipid-transfer protein 1 [Oryza sativa Japonica Group]	2	120	12.1	8.92	1	120	12.1	8.92	0.500
410	BAS91062.1	Os04g0620700, partial [Oryza sativa Japonica Group]	2	569	60.5	4.77	10	569	60.5	4.77	5.000
411	BAT16529.1	Os12g0247533 [Oryza sativa Japonica Group]	4	293	32.6	4.87	2	293	32.6	4.87	0.500
412	XP_015611898.1	polyadenylate-binding protein 2 [Oryza sativa Japonica Group]	3	662	71.6	6.83	1	662	71.6	6.83	0.333
413	ABF98863.1	Cysteine synthase, putative, expressed [Oryza sativa Japonica Group]	3	259	27.2	5.97	3	259	27.2	5.97	1.000

414	XP_015630542.1	T-complex protein 1 subunit theta [Oryza sativa Japonica Group]	3	546	58.7	5.43	4	546	58.7	5.43	1.333
415	XP_015651037.1	grpE protein homolog 2, mitochondrial isoform X2 [Oryza sativa Japonica Group]	2	311	33.7	5.35	2	311	33.7	5.35	1.000
416	EEC81568.1	hypothetical protein OsI_25013 [Oryza sativa Indica Group]	2	84	9.5	5.68	5	84	9.5	5.68	2.500
417	CAJ86144.1	H0701F11.10 [Oryza sativa]	2	316	33.5	5.40	1	316	33.5	5.40	0.500
418	XP_015650677.1	acyl carrier protein 3, chloroplastic [Oryza sativa Japonica Group]	2	138	14.5	5.12	2	138	14.5	5.12	1.000
419	AAB53367.1	pathogenesis-related thaumatin-like protein [Oryza sativa]	2	181	19.2	4.79	2	181	19.2	4.79	1.000
420	XP_015637440.1	gamma-glutamyl hydrolase 2 [Oryza sativa Japonica Group]	2	337	36.9	8.35	5	337	36.9	8.35	2.500
421	EEC79354.1	hypothetical protein OsI_20231 [Oryza sativa Indica Group]	1	95	10.5	9.60	4	95	10.5	9.60	4.000
422	BAD09617.1	putative aminopeptidase N [Oryza sativa Japonica Group]	1	875	98.0	5.66	3	875	98.0	5.66	3.000
423	XP_015623205.1	uncharacterized protein LOC4330678 [Oryza sativa Japonica Group]	2	109	12.3	8.12	3	109	12.3	8.12	1.500
424	EAY84337.1	hypothetical protein OsI_05714 [Oryza sativa Indica Group]	2	131	13.8	10.24	1	131	13.8	10.24	0.500
425	XP_015625800.1	proteasome subunit beta type-6 [Oryza sativa Japonica Group]	3	246	26.3	5.78	3	246	26.3	5.78	1.000
426	BAT03386.1	Os07g0695800, partial [Oryza sativa Japonica Group]	1	504	57.7	7.42	3	504	57.7	7.42	3.000
427	BAS73238.1	Os01g0624000, partial [Oryza sativa Japonica Group]	2	405	44.9	7.08	1	405	44.9	7.08	0.500
428	EAY92196.1	hypothetical protein OsI_13914 [Oryza sativa Indica Group]	1	293	29.6	10.76	2	293	29.6	10.76	2.000
429	XP_015641204.1	monocopper oxidase-like protein SKU5 [Oryza sativa Japonica Group]	2	593	65.7	6.55	1	593	65.7	6.55	0.500
430	CAJ86138.1	H0701F11.4 [Oryza sativa]	2	196	22.0	5.91	1	196	22.0	5.91	0.500
431	XP_015613005.1	uncharacterized protein LOC4348196 [Oryza sativa Japonica Group]	1	386	42.6	5.02	4	386	42.6	5.02	4.000
432	EEC73887.1	hypothetical protein OsI_08681 [Oryza sativa Indica Group]	2	325	36.1	6.79	2	325	36.1	6.79	1.000
433	XP_015635047.1	gibberellin-regulated protein 14 [Oryza sativa Japonica Group]	2	105	11.1	8.37	5	105	11.1	8.37	2.500
434	CAD48598.1	gamma-glutamylcysteine synthetase [Oryza sativa Japonica Group]	2	438	50.3	5.43	3	438	50.3	5.43	1.500
435	AAT76418.1	expressed protein [Oryza sativa Japonica Group]	1	190	21.0	5.66	2	190	21.0	5.66	2.000
436	BAD17079.1	putative phi-1 [Oryza sativa Japonica Group]	1	316	32.8	8.72	3	316	32.8	8.72	3.000

437	EAY75654.1	hypothetical protein OsI_03559 [Oryza sativa Indica Group]	2	332	36.0	6.70	4	332	36.0	6.70	2.000
438	CAC39056.1	putative protein [Oryza sativa]	2	227	24.6	4.50	2	227	24.6	4.50	1.000
439	BAD23595.1	putative eukaryotic translation initiation factor 5 [Oryza sativa Japonica Group]	1	333	36.0	5.50	1	333	36.0	5.50	1.000
440	XP_015622612.1	sucrose-phosphatase 2 [Oryza sativa Japonica Group]	2	423	47.2	5.82	2	423	47.2	5.82	1.000
441	EEE54097.1	hypothetical protein OsJ_00845 [Oryza sativa Japonica Group]	2	161	17.3	7.50	2	161	17.3	7.50	1.000
442	XP_015647517.1	uncharacterized protein LOC4343118 isoform X2 [Oryza sativa Japonica Group]	1	543	58.4	6.06	6	543	58.4	6.06	6.000
443	CAH66571.1	OSIGBa0148P16.5 [Oryza sativa]	2	389	41.3	5.81	2	389	41.3	5.81	1.000
444	AAM03044.1	putative dihydroorotate dehydrogenase, partial [Oryza sativa Japonica Group]	1	201	22.0	6.67	1	201	22.0	6.67	1.000
445	BAT12969.1	Os11g0183900, partial [Oryza sativa Japonica Group]	1	294	31.9	9.25	6	294	31.9	9.25	6.000
446	XP_015650211.1	succinate dehydrogenase [ubiquinone] iron-sulfur subunit 1, mitochondrial isoform X2 [Oryza sativa Japonica Group]	2	281	31.1	8.48	3	281	31.1	8.48	1.500
447	BAF22073.1	Os07g0592000 [Oryza sativa Japonica Group]	1	102	10.7	8.37	1	102	10.7	8.37	1.000
448	CAD41093.2	OSJNBb0011N17.10 [Oryza sativa Japonica Group]	2	230	24.6	5.12	1	230	24.6	5.12	0.500
449	EAZ00738.1	hypothetical protein OsI_22765 [Oryza sativa Indica Group]	1	81	8.7	6.32	3	81	8.7	6.32	3.000
450	XP_015615680.1	probable purple acid phosphatase 20 [Oryza sativa Japonica Group]	1	439	47.8	6.33	1	439	47.8	6.33	1.000
451	NP_001147283.1	40S ribosomal protein S28 [Zea mays]	1	65	7.5	11.17	1	65	7.5	11.17	1.000
452	XP_006649713.1	PREDICTED: NHP2-like protein 1 [Oryza brachyantha]	1	128	13.9	7.12	3	128	13.9	7.12	3.000
453	EAZ37366.1	hypothetical protein OsJ_21705 [Oryza sativa Japonica Group]	1	535	59.2	6.01	3	535	59.2	6.01	3.000
454	AAX55895.1	aci-reductone dioxygenase-like protein [Oryza sativa Japonica Group]	1	198	23.5	5.20	1	198	23.5	5.20	1.000
455	XP_015633082.1	non-specific lipid transfer protein-like 1 isoform X2 [Oryza sativa Japonica Group]	1	178	16.9	8.25	1	178	16.9	8.25	1.000
456	XP_015642583.1	probable LRR receptor-like serine/threonine-protein kinase At1g34110 [Oryza sativa Japonica Group]	1	434	45.0	4.77	1	434	45.0	4.77	1.000
457	EAZ17077.1	hypothetical protein OsJ_32575 [Oryza sativa Japonica Group]	1	308	34.6	6.52	1	308	34.6	6.52	1.000
458	EAY86155.1	hypothetical protein OsI_07530 [Oryza sativa Indica Group]	1	301	33.5	5.57	5	301	33.5	5.57	5.000
459	XP_015651422.1	uncharacterized protein LOC4347568 [Oryza sativa Japonica Group]	1	158	17.1	7.37	2	158	17.1	7.37	2.000
460	BAT01828.1	Os07g0523300, partial [Oryza sativa Japonica Group]	1	37	4.2	9.60	1	37	4.2	9.60	1.000

368	XP_015650117.1	70 kDa peptidyl-prolyl isomerase [Oryza sativa Japonica Group]	4	580	64.1	5.24	5	580	64.1	5.24	1.250
369	EEE58349.1	hypothetical protein OsJ_09473 [Oryza sativa Japonica Group]	4	505	55.8	5.74	3	505	55.8	5.74	0.750
370	XP_015622173.1	ubiquitin-fold modifier 1 [Oryza sativa Japonica Group]	3	102	10.4	9.61	2	102	10.4	9.61	0.667
371	XP_015645047.1	uncharacterized GPI-anchored protein At5g19250 [Oryza sativa Japonica Group]	3	194	20.7	5.43	2	194	20.7	5.43	0.667
372	ABR26120.1	nucleosome chromatin assembly protein, partial [Oryza sativa Indica Group]	4	243	27.5	4.42	2	243	27.5	4.42	0.500
373	EEE70144.1	hypothetical protein OsJ_30190 [Oryza sativa Japonica Group]	2	216	23.5	7.69	2	216	23.5	7.69	1.000
374	CAH67498.1	H0306B06.13 [Oryza sativa]	5	221	22.5	8.97	5	221	22.5	8.97	1.000
375	EAY81553.1	hypothetical protein OsI_36719 [Oryza sativa Indica Group]	3	148	16.0	7.93	2	148	16.0	7.93	0.667
376	XP_015615882.1	uncharacterized protein LOC4349779 [Oryza sativa Japonica Group]	5	146	15.8	5.05	9	146	15.8	5.05	1.800
377	XP_015643260.1	fructose-bisphosphate aldolase 3, chloroplastic [Oryza sativa Japonica Group]	3	388	42.0	8.62	1	388	42.0	8.62	0.333
378	XP_015628800.1	defensin-like protein [Oryza sativa Japonica Group]	3	81	8.8	8.51	5	81	8.8	8.51	1.667
379	BAT08543.1	Os09g0469400 [Oryza sativa Japonica Group]	3	339	37.7	5.07	1	339	37.7	5.07	0.333
380	AAC04834.1	germin-like protein 3, partial [Oryza sativa Japonica Group]	3	181	19.6	8.72	1	181	19.6	8.72	0.333
381	XP_015621646.1	isoflavone reductase homolog IRL [Oryza sativa Japonica Group]	4	314	33.5	6.05	10	314	33.5	6.05	2.500
382	EAZ29987.1	hypothetical protein OsJ_14046 [Oryza sativa Japonica Group]	3	319	34.3	6.65	1	319	34.3	6.65	0.333
383	BAS77791.1	Os02g0232400, partial [Oryza sativa Japonica Group]	2	284	31.6	9.36	4	284	31.6	9.36	2.000
384	XP_015627578.1	60S ribosomal protein L6-3 [Oryza sativa Japonica Group]	4	219	24.2	10.11	3	219	24.2	10.11	0.750
385	XP_015624643.1	cysteine proteinase 1 [Oryza sativa Japonica Group]	2	373	40.6	6.47	1	373	40.6	6.47	0.500
386	EAZ01248.1	hypothetical protein OsI_23273 [Oryza sativa Indica Group]	3	164	17.6	5.80	6	164	17.6	5.80	2.000
387	EEC73818.1	hypothetical protein OsI_08537 [Oryza sativa Indica Group]	2	443	48.6	5.36	3	443	48.6	5.36	1.500
388	XP_015628599.1	MD-2-related lipid-recognition protein ROSY1 [Oryza sativa Japonica Group]	3	156	16.9	6.06	5	156	16.9	6.06	1.667
389	ABA93514.1	hypothetical protein LOC_Os11g26970 [Oryza sativa Japonica Group]	5	111	12.9	8.16	14	111	12.9	8.16	2.800
390	BAS85556.1	Os03g0655700, partial [Oryza sativa Japonica Group]	4	198	21.3	5.66	6	198	21.3	5.66	1.500
391	XP_015650252.1	NADP-dependent glyceraldehyde-3-phosphate dehydrogenase [Oryza sativa Japonica Group]	3	499	53.3	7.01	9	499	53.3	7.01	3.000

461	AAT77374.1	putative beta-N-acetylhexosaminidase [Oryza sativa Japonica Group]	1	527	58.6	6.09	2	527	58.6	6.09	2.000
462	EAZ39503.1	hypothetical protein OsJ_23938 [Oryza sativa Japonica Group]	1	501	54.2	8.13	5	501	54.2	8.13	5.000
463	BAS84077.1	Os03g0335300, partial [Oryza sativa Japonica Group]	1	458	51.2	5.69	3	458	51.2	5.69	3.000
464	A2XAZ3.1	RecName: Full=Alcohol dehydrogenase class-3; AltName: Full=Alcohol dehydrogenase class-III; AltName: Full=Glutathione-dependent formaldehyde dehydrogenase; Short=FALDH; Short=FDH; Short=GSH-FDH; AltName: Full=S-(hydroxymethyl)glutathione dehydrogenase	1	381	40.8	7.17	3	381	40.8	7.17	3.000
465	BAD87139.1	glutathione S-transferase-like [Oryza sativa Japonica Group]	1	65	7.5	5.01	1	65	7.5	5.01	1.000
466	AAL65399.1	3-ketoacyl-CoA thiolase, partial [Oryza sativa Japonica Group]	1	177	18.6	8.27	3	177	18.6	8.27	3.000
467	BAS87105.1	Os03g0822000 [Oryza sativa Japonica Group]	1	115	12.6	10.84	2	115	12.6	10.84	2.000
468	NP_001356196.1	uncharacterized protein LOC100282946 [Zea mays]	1	62	6.9	12.03	3	62	6.9	12.03	3.000
469	XP_015626102.1	glutamine synthetase cytosolic isozyme 1-1 [Oryza sativa Japonica Group]	1	356	39.2	5.73	1	356	39.2	5.73	1.000
470	XP_015645264.1	probable glutathione S-transferase GSTU1 [Oryza sativa Japonica Group]	1	232	25.8	5.12	2	232	25.8	5.12	2.000
471	EAZ30807.1	hypothetical protein OsJ_14873 [Oryza sativa Japonica Group]	1	266	27.7	6.81	1	266	27.7	6.81	1.000
472	AAD39987.1	small zinc finger-like protein [Oryza sativa]	1	84	9.0	5.25	2	84	9.0	5.25	2.000
473	XP_015620971.1	probable calcium-binding protein CML16 [Oryza sativa Japonica Group]	1	181	19.3	4.59	3	181	19.3	4.59	3.000
474	BAD61191.1	hypothetical protein [Oryza sativa Japonica Group]	1	72	7.6	11.94	2	72	7.6	11.94	2.000
475	EAZ20562.1	hypothetical protein OsJ_36171 [Oryza sativa Japonica Group]	1	411	47.2	6.21	1	411	47.2	6.21	1.000
476	BAS89786.1	Os04g0485300, partial [Oryza sativa Japonica Group]	1	56	6.3	5.74	1	56	6.3	5.74	1.000
477	BAF14255.1	Os04g0270100, partial [Oryza sativa Japonica Group]	1	444	49.7	7.05	2	444	49.7	7.05	2.000

All salt-soluble proteins detected in both MSB and WSB were listed in descending order of PSM of MSB. The salt-soluble proteins detected in both MSB and NSB were 477 species.
